# Evolutionary convergence and divergence of hippocampal cytoarchitecture between rodents and primates revealed by single-cell spatial transcriptomics

**DOI:** 10.1093/nsr/nwaf595

**Published:** 2026-01-02

**Authors:** Tianyi Fei, Yu Dong, Xiaojuan Gou, Jiahao Zhang, Guangling Wang, Nini Yuan, Zhenkun Zhuang, Duoyuan Chen, Zihan Wu, Lijie Zhan, Ning Liang, Nicholas Mitsios, Jingfang Pan, Yafeng Zhan, Mengdi Cao, Ying Xia, Haotian Yan, Xiaojia Zhu, Yuzhe Huang, Shishuo Chen, Hao Yang, Liqin Gu, He Wang, Yanqing Zhong, Qian Yu, Yingjie An, Ruiqi Wang, Shenyu Li, Jingjing Song, Wanqiu Zhou, Shuzhen Zhang, Biyu Ren, Yanbing Lu, Wenqun Ding, Shuting Li, Huateng Cao, Junkai Lin, Yannong Dou, Xuyin Jiang, Chun Xie, Yan Li, Xing Tan, Yuanfang Xu, Xinxiang Song, Huanhuan Li, Mengni Chen, Shiwen Wang, Mingyuan Zheng, Jiao Fang, Ruiyi Zhang, Qinwen Chai, Mingli Wang, Li Li, Taosha Gao, Liuqin Qian, Chenxi Jin, Jiawen Huang, Wei Deng, Minghai Li, Chao Li, Zhiyong Liu, Zhifeng Liang, Shengjin Xu, Mathias Uhlén, Mu-Ming Poo, Jianhua Yao, Shiping Liu, Cirong Liu, Zhiming Shen, Yan-Gang Sun, Chengyu T Li, Jan Mulder, Gang Cao, Yidi Sun, Chun Xu

**Affiliations:** Institute of Neuroscience, Center for Excellence in Brain Science and Intelligence Technology, Chinese Academy of Sciences, Shanghai 200031, China; School of Life Sciences and Technology, ShanghaiTech University, Shanghai 201210, China; Institute of Neuroscience, Center for Excellence in Brain Science and Intelligence Technology, Chinese Academy of Sciences, Shanghai 200031, China; Lingang Laboratory, Shanghai 200031, China; ENT Institute and Otorhinolaryngology Department of Eye & ENT Hospital, State Key Laboratory of Medical Neurobiology and MOE Frontiers Center for Brain Science, Fudan University, Shanghai 200031, China; State Key Laboratory of Agricultural Microbiology, Hubei Hongshan Laboratory, Huazhong Agricultural University, Wuhan 430070, China; College of Veterinary Medicine, Huazhong Agricultural University, Wuhan 430070, China; Key Laboratory of Zoonoses, Ministry of Agriculture and Rural Affairs, College of Veterinary Medicine, South China Agricultural University, Guangzhou 510642, China; Institute of Neuroscience, Center for Excellence in Brain Science and Intelligence Technology, Chinese Academy of Sciences, Shanghai 200031, China; Institute of Neuroscience, Center for Excellence in Brain Science and Intelligence Technology, Chinese Academy of Sciences, Shanghai 200031, China; BGI Research, Shenzhen 518083, China; BGI Research, Shenzhen 518083, China; Tencent AI Lab, Shenzhen 518057, China; Institute of Neuroscience, Center for Excellence in Brain Science and Intelligence Technology, Chinese Academy of Sciences, Shanghai 200031, China; BGI Research, Shenzhen 518083, China; Department of Neuroscience, Karolinska Institutet, Stockholm 17177, Sweden; Department of Neuroscience, Karolinska Institutet, Stockholm 17177, Sweden; BGI Research, Hangzhou 310030, China; Institute of Neuroscience, Center for Excellence in Brain Science and Intelligence Technology, Chinese Academy of Sciences, Shanghai 200031, China; Institute of Neuroscience, Center for Excellence in Brain Science and Intelligence Technology, Chinese Academy of Sciences, Shanghai 200031, China; Institute of Neuroscience, Center for Excellence in Brain Science and Intelligence Technology, Chinese Academy of Sciences, Shanghai 200031, China; Institute of Neuroscience, Center for Excellence in Brain Science and Intelligence Technology, Chinese Academy of Sciences, Shanghai 200031, China; Institute of Neuroscience, Center for Excellence in Brain Science and Intelligence Technology, Chinese Academy of Sciences, Shanghai 200031, China; Shanghai Center for Brain Science and Brain-Inspired Technology, Shanghai 201602, China; School of Life Sciences and Technology, ShanghaiTech University, Shanghai 201210, China; Institute of Neuroscience, Center for Excellence in Brain Science and Intelligence Technology, Chinese Academy of Sciences, Shanghai 200031, China; University of Chinese Academy of Sciences, Beijing 100049, China; Institute of Neuroscience, Center for Excellence in Brain Science and Intelligence Technology, Chinese Academy of Sciences, Shanghai 200031, China; Institute of Neuroscience, Center for Excellence in Brain Science and Intelligence Technology, Chinese Academy of Sciences, Shanghai 200031, China; Institute of Neuroscience, Center for Excellence in Brain Science and Intelligence Technology, Chinese Academy of Sciences, Shanghai 200031, China; Institute of Neuroscience, Center for Excellence in Brain Science and Intelligence Technology, Chinese Academy of Sciences, Shanghai 200031, China; Institute of Neuroscience, Center for Excellence in Brain Science and Intelligence Technology, Chinese Academy of Sciences, Shanghai 200031, China; Institute of Neuroscience, Center for Excellence in Brain Science and Intelligence Technology, Chinese Academy of Sciences, Shanghai 200031, China; Institute of Neuroscience, Center for Excellence in Brain Science and Intelligence Technology, Chinese Academy of Sciences, Shanghai 200031, China; Institute of Neuroscience, Center for Excellence in Brain Science and Intelligence Technology, Chinese Academy of Sciences, Shanghai 200031, China; Institute of Neuroscience, Center for Excellence in Brain Science and Intelligence Technology, Chinese Academy of Sciences, Shanghai 200031, China; Institute of Neuroscience, Center for Excellence in Brain Science and Intelligence Technology, Chinese Academy of Sciences, Shanghai 200031, China; Institute of Neuroscience, Center for Excellence in Brain Science and Intelligence Technology, Chinese Academy of Sciences, Shanghai 200031, China; Institute of Neuroscience, Center for Excellence in Brain Science and Intelligence Technology, Chinese Academy of Sciences, Shanghai 200031, China; Institute of Neuroscience, Center for Excellence in Brain Science and Intelligence Technology, Chinese Academy of Sciences, Shanghai 200031, China; Institute of Neuroscience, Center for Excellence in Brain Science and Intelligence Technology, Chinese Academy of Sciences, Shanghai 200031, China; Institute of Neuroscience, Center for Excellence in Brain Science and Intelligence Technology, Chinese Academy of Sciences, Shanghai 200031, China; Institute of Neuroscience, Center for Excellence in Brain Science and Intelligence Technology, Chinese Academy of Sciences, Shanghai 200031, China; Institute of Neuroscience, Center for Excellence in Brain Science and Intelligence Technology, Chinese Academy of Sciences, Shanghai 200031, China; Institute of Neuroscience, Center for Excellence in Brain Science and Intelligence Technology, Chinese Academy of Sciences, Shanghai 200031, China; Institute of Neuroscience, Center for Excellence in Brain Science and Intelligence Technology, Chinese Academy of Sciences, Shanghai 200031, China; Institute of Neuroscience, Center for Excellence in Brain Science and Intelligence Technology, Chinese Academy of Sciences, Shanghai 200031, China; Institute of Neuroscience, Center for Excellence in Brain Science and Intelligence Technology, Chinese Academy of Sciences, Shanghai 200031, China; Institute of Neuroscience, Center for Excellence in Brain Science and Intelligence Technology, Chinese Academy of Sciences, Shanghai 200031, China; Institute of Neuroscience, Center for Excellence in Brain Science and Intelligence Technology, Chinese Academy of Sciences, Shanghai 200031, China; Institute of Neuroscience, Center for Excellence in Brain Science and Intelligence Technology, Chinese Academy of Sciences, Shanghai 200031, China; Institute of Neuroscience, Center for Excellence in Brain Science and Intelligence Technology, Chinese Academy of Sciences, Shanghai 200031, China; Institute of Neuroscience, Center for Excellence in Brain Science and Intelligence Technology, Chinese Academy of Sciences, Shanghai 200031, China; Institute of Neuroscience, Center for Excellence in Brain Science and Intelligence Technology, Chinese Academy of Sciences, Shanghai 200031, China; Institute of Neuroscience, Center for Excellence in Brain Science and Intelligence Technology, Chinese Academy of Sciences, Shanghai 200031, China; Institute of Neuroscience, Center for Excellence in Brain Science and Intelligence Technology, Chinese Academy of Sciences, Shanghai 200031, China; Institute of Neuroscience, Center for Excellence in Brain Science and Intelligence Technology, Chinese Academy of Sciences, Shanghai 200031, China; Institute of Neuroscience, Center for Excellence in Brain Science and Intelligence Technology, Chinese Academy of Sciences, Shanghai 200031, China; Institute of Neuroscience, Center for Excellence in Brain Science and Intelligence Technology, Chinese Academy of Sciences, Shanghai 200031, China; School of Life Sciences and Technology, ShanghaiTech University, Shanghai 201210, China; Institute of Neuroscience, Center for Excellence in Brain Science and Intelligence Technology, Chinese Academy of Sciences, Shanghai 200031, China; Institute of Neuroscience, Center for Excellence in Brain Science and Intelligence Technology, Chinese Academy of Sciences, Shanghai 200031, China; Institute of Neuroscience, Center for Excellence in Brain Science and Intelligence Technology, Chinese Academy of Sciences, Shanghai 200031, China; Institute of Neuroscience, Center for Excellence in Brain Science and Intelligence Technology, Chinese Academy of Sciences, Shanghai 200031, China; Institute of Neuroscience, Center for Excellence in Brain Science and Intelligence Technology, Chinese Academy of Sciences, Shanghai 200031, China; Institute of Neuroscience, Center for Excellence in Brain Science and Intelligence Technology, Chinese Academy of Sciences, Shanghai 200031, China; Institute of Neuroscience, Center for Excellence in Brain Science and Intelligence Technology, Chinese Academy of Sciences, Shanghai 200031, China; Institute of Neuroscience, Center for Excellence in Brain Science and Intelligence Technology, Chinese Academy of Sciences, Shanghai 200031, China; Institute of Neuroscience, Center for Excellence in Brain Science and Intelligence Technology, Chinese Academy of Sciences, Shanghai 200031, China; Institute of Neuroscience, Center for Excellence in Brain Science and Intelligence Technology, Chinese Academy of Sciences, Shanghai 200031, China; Institute of Neuroscience, Center for Excellence in Brain Science and Intelligence Technology, Chinese Academy of Sciences, Shanghai 200031, China; Department of Neuroscience, Karolinska Institutet, Stockholm 17177, Sweden; Science for Life Laboratory, KTH-Royal Institute of Technology, Solna 17121, Sweden; Institute of Neuroscience, Center for Excellence in Brain Science and Intelligence Technology, Chinese Academy of Sciences, Shanghai 200031, China; Shanghai Center for Brain Science and Brain-Inspired Technology, Shanghai 201602, China; Tencent AI Lab, Shenzhen 518057, China; BGI Research, Shenzhen 518083, China; Institute of Neuroscience, Center for Excellence in Brain Science and Intelligence Technology, Chinese Academy of Sciences, Shanghai 200031, China; Key Laboratory of Genetic Evolution and Animal Models, Chinese Academy of Sciences, Shanghai 200031, China; Institute of Neuroscience, Center for Excellence in Brain Science and Intelligence Technology, Chinese Academy of Sciences, Shanghai 200031, China; Institute of Neuroscience, Center for Excellence in Brain Science and Intelligence Technology, Chinese Academy of Sciences, Shanghai 200031, China; University of Chinese Academy of Sciences, Beijing 100049, China; National Key Laboratory of Brain Cognition and Brain-inspired Intelligence Technology, Chinese Academy of Sciences, Shanghai 200031, China; Lingang Laboratory, Shanghai 200031, China; Department of Neuroscience, Karolinska Institutet, Stockholm 17177, Sweden; Shenzhen Institute of Advanced Technology, Shenzhen 518055, China; Faculty of Life and Health Sciences, Shenzhen University of Advanced Technology, Shenzhen 518107, China; Institute of Neuroscience, Center for Excellence in Brain Science and Intelligence Technology, Chinese Academy of Sciences, Shanghai 200031, China; Key Laboratory of Genetic Evolution and Animal Models, Chinese Academy of Sciences, Shanghai 200031, China; Institute of Neuroscience, Center for Excellence in Brain Science and Intelligence Technology, Chinese Academy of Sciences, Shanghai 200031, China; University of Chinese Academy of Sciences, Beijing 100049, China; National Key Laboratory of Brain Cognition and Brain-inspired Intelligence Technology, Chinese Academy of Sciences, Shanghai 200031, China; China-Hungary Belt-and-Road Joint Laboratory on Brain Science, Shanghai 200031, China

**Keywords:** spatial transcriptome, hippocampus, evolution, rodent, primate

## Abstract

The hippocampus comprises subregions of distinct cell types critical for memory and cognition, but their gene expression profiles and spatial distribution patterns remain to be clarified. Using single-cell spatial transcriptomic analysis and single-nucleus RNA sequencing, we obtained transcriptome-based atlases for the macaque, marmoset and mouse hippocampus. Cross-species comparison revealed primate- and lamina-specific glutamatergic cell types in the subicular complex, as well as enrichment of *VIP*-expressing GABAergic cells from mice to primates, including humans. Furthermore, we found reduced transcriptomic differences between CA3 and CA4 subregions and distinct longitudinal distributions of various cell types and expression of ion-channel genes, correlated with differences in electrophysiological properties of CA3, CA4 and CA1 neurons revealed by slice recording from marmosets and mice. Collectively, this cross-species study provides a molecular and cellular basis for understanding the evolution and function of the hippocampus.

## INTRODUCTION

The hippocampus is an evolutionarily conserved brain structure in vertebrate species [1,2] and is essential for memory [3], cognition [4], stress [5] and emotion [5,6] in mammals. The mammalian hippocampus comprises anatomically and functionally distinct subregions including the dentate gyrus (DG), the *cornu ammonis* (CA) fields and the subicular complex, with interconnections among them [7,8]. In the mammalian hippocampus, the CA field is divided into CA1, CA2, CA3 and CA4 subregions and the subiculum complex into the prosubiculum (ProS), subiculum proper (Sub), pre-subiculum (PreS), post-subiculum (PostS) and para-subiculum (ParaS), based on their distinct cytoarchitecture and connectivity [1,9–11]. There remains uncertainty in the boundaries for CA fields, as well as for subicular subregions [11–13], although there is evidence supporting the existence of these subregions based on receptor mapping [12] and *in situ* hybridization [14]. The heterogeneity in the connectivity and function along the hippocampal longitudinal axis [2,15,16] may be related to distinct gene expression profiles [17–19], but transcriptome-defined cell types and their longitudinal distributions remain to be clarified, particularly for the primate hippocampus. Thus, there is a need for systematic and comprehensive mapping of gene expression patterns in various hippocampal subregions and longitudinal locations. Gene expression patterns within and across hippocampal subregions could also provide the neuronal markers for studying region-specific connectivity and functions [19].

Recent advances in single-cell RNA sequencing have revealed substantial molecular and functional diversity of hippocampal cells [20–23], but the precise spatial origin of identified cells remains unclear. Spatial transcriptome sequencing methods now enable spatial mapping of gene expression in the brain of rodents and primates [24–27]. Together with single-cell sequencing, the latest Stereo-seq method [25] could provide more comprehensive cell-type classifications and their spatial distribution. Moreover, cross-species comparison of gene expression patterns may enable the characterization of evolutionary changes of cell types [28–30] and their subregion specialization.

In this study, we combined Stereo-seq and single-nucleus RNA sequencing (snRNA-seq) to classify the cell types and map their spatial distributions for the entire hippocampus of macaques, marmosets and mice. We have identified species-dependent profiles and composition of neuronal cell types, lamina- and primate-specific glutamatergic cell types in the subicular complex, which was consistent with our analysis of human spatial transcriptome data. We have further revealed the heterogeneity in cell-type and gene-expression spatial distributions along multiple hippocampal axes. Electrophysiological recording from acute hippocampal slices of mice and marmosets validated the heterogeneity in the physiological properties of hippocampal neurons along the longitudinal axis, as well as between CA3 and CA3 subregions. Importantly, we obtained comprehensive atlases for the spatial transcriptome in the hippocampus with single-cell resolution for all three species (accessible online at https://digital-brain.cn/cross-species/hipp/). Our results provide a molecular and cellular basis for understanding the evolution and function of various subregions and diverse cell types of the hippocampus.

## RESULTS

### Spatial transcriptome-defined hippocampal subregions across species

**Figure 1.**
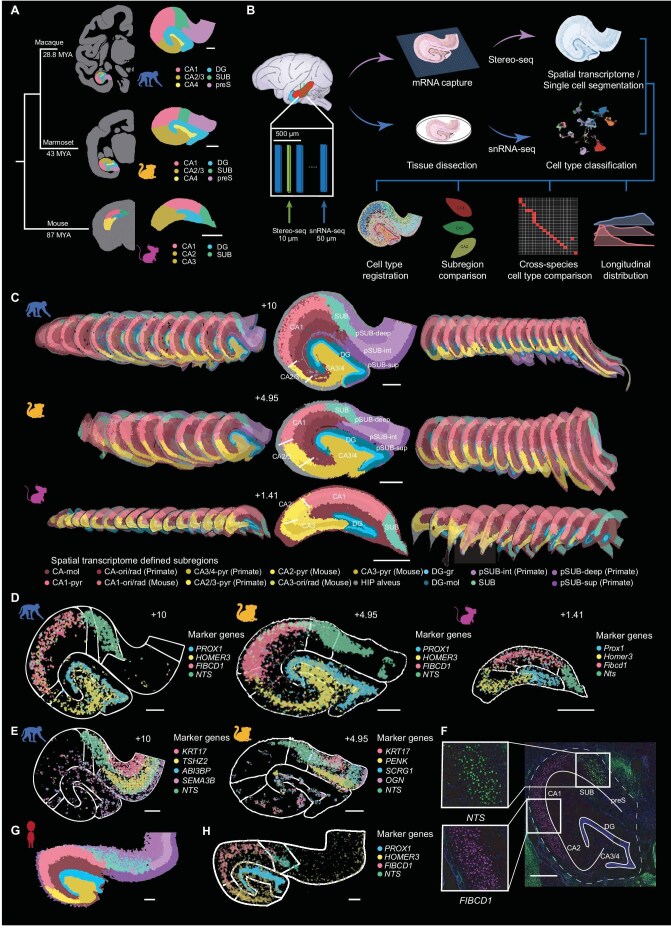
Hippocampal subregions defined by spatial transcriptomic profiles. (A) Left, a phylogenetic tree of macaque, marmoset and mouse (in million years ago, MYA). Right, enlarged hippocampus of each species and color-coded subregions defined by a conventional histology-based atlas. (B) The procedure of data acquisition and analysis based on Stereo-seq and snRNA-seq of the macaque, marmoset and mouse hippocampus. The box illustrates consecutive coronal sections for Stereo-seq (green) and snRNA-seq (blue) analyses. Cell type classifications based on snRNA-seq data and single-cell transcriptome maps based on Stereo-seq data were used for defining transcriptome-based subregions, cross-species comparison of transcriptomic profiles and cell types, and longitudinal profiles in cell type distribution and gene expression. (C) Hippocampal subregions of the three species (macaque, marmoset, mouse) defined by unsupervised clustering analysis of Stereo-seq data. The sections were presented along the longitudinal axis, with one section (EBZ coordinate shown) enlarged in the frontal view. The subregions are color-coded with annotations (see text) shown below. (D) Spatial expression patterns of example marker genes for hippocampal subregions conserved across species: *PROX1* for DG, *FIBCD1* for CA1, *HOMER3* for CA3/4 and *NTS* for subiculum (SUB). Contours mark conventional histology-defined subregions. (E) Spatial visualization of genes marking laminar structures in the primate subicular complex. Contours mark conventional histology-defined subregions. (F) FISH validation for the expression of SUB and CA1 marker genes *FIBCD1* and *NTS*, respectively. (G) Human hippocampal subregions defined by unsupervised clustering analysis of Stereo-seq data. Subregions are shown in the same color codes as those in C. (H) Spatial expression patterns of example marker genes for hippocampal subregions conserved across species in human sections: *PROX1* for DG, *FIBCD1* for CA1, *HOMER3* for CA3/4 and *NTS* for subiculum (SUB). Scale bars, 1 mm.

To reveal the hippocampal subregions with distinct gene expression profiles, we used a spatial transcriptome sequencing method (Stereo-seq) [24,25] to systematically map spatial patterns of gene expression in the macaque, marmoset and mouse hippocampus (Fig. 1A). We collected more than 30 coronal sections of 10-μm thickness along the anterior–posterior axis of each hippocampus for Stereo-seq (macaque, 30 sections, 0.5 mm spacing; marmoset, 35 sections, 0.25 mm spacing; mouse, 33 sections, 0.1 mm spacing; see details in Table S1), harvested single nuclei from adjacent sections (50 μm thick) for snRNA-seq, and finally integrated the snRNA-seq and Stereo-seq data to obtain a cellular spatial transcriptome map for various cell types in all sections (Fig. 1B). For each section, we first analyzed Stereo-seq data by the unsupervised spatial clustering method and then defined hippocampal subregions by overall spatial transcriptome profiles (Fig. 1C; see Materials and Methods). Jaccard similarity analysis showed that various transcriptome-defined subregions were highly consistent across hippocampal sections (Fig. S1A). Similar transcriptome-defined subregions were found for hippocampal sections from animal replicates in each species (Fig. S1B).

We next compared our transcriptome-based subregions with those defined in conventional histology-based atlases [31–34]. We found that the pyramidal cell layers of different subregions in existing atlases matched well spatially with those defined by transcriptome profiles (Fig. S1C). We thus annotated spatial transcriptomic clusters corresponding to the *stratum (str.) pyramidale* in CA1, CA2, CA3, the granular cell layer in DG, and the pro-subiculum and subiculum proper as CA1 pyramidale (CA1-pyr), CA2-pyr, CA3-pyr, DG-gr and SUB, respectively (Fig. 1C). For primate hippocampal sections, some spatial transcriptome clusters were annotated as CA2/3-pyr and CA3/4-pyr because they overlapped between adjacent subregions. Similarly, the canonical *str. lacunosum-moleculare* in CA1, CA2 and CA3 together showed a common transcriptomic profile and were thus annotated as CA-mol, whereas the outer part of the DG molecular layer was annotated as DG-mol. Furthermore, conventional *str. radiatum* and *str. oriens* in CA1 generally had a low number of neurons and low gene expression as a whole, and were annotated as CA1-ori/rad. The same was found for *str. radiatum* and *str. oriens* in CA3 (annotated as CA3-ori/rad) (Fig. 1C and Fig. S1C). Notably, the pre- and para-subiculum (pSUB) in primates together corresponded to three transcriptomic profiles with laminar organization and were annotated as deep, intermediate and superficial layers of pSUB (termed pSUB-deep, pSUB-int and pSUB-sup), respectively (Fig. 1C and Fig. S1D). As shown later, these subregions defined by spatial transcriptome profiles correspond to distinct distribution of transcriptome-defined cell types, which provide the molecular and cellular basis for hippocampal subdivision and functional specification. The spatial transcriptome-based annotations for macaque and marmoset subregions were mapped onto high-resolution brain templates generated from functional magnetic resonance imaging (fMRI) data [35] (see integrative atlases at https://digital-brain.cn/cross-species/hipp).

We next examined the similarity between transcriptomic profiles of corresponding hippocampal subregions in macaques, marmosets and mice (Fig. S1E), and identified numerous evolutionarily conserved gene expression patterns for homologous subregions such as *PROX1* in DG, *HOMER3* in CA3/4, *F1BCD1* in CA1, and *NTS* in SUB (Fig. 1D and Fig. S1F; see marker genes in Table S2). Furthermore, the pSUB-deep transcriptomic profile of macaques and marmosets shared a common marker gene, *KRT17* (Fig. 1E). Finally, we validated the selective expression of *F1BCD1* in CA1 and *NTS* in the subiculum by fluorescence *in situ* hybridization (FISH) assay in hippocampal sections of marmosets (Fig. 1F). Finally, we obtained human spatial transcriptome data using the Stereo-seq method, and validated marker genes for homologous subregions such as DG, CA3/4, CA1 and SUB, and the laminar organization of the pSUB subregion (Fig. S1G–S1I). Taken together, these results provide spatial transcriptome-based hippocampal subdivision and the molecular basis for homologous hippocampal subregions across species.

### Spatial distribution of cell types based on Stereo-seq and snRNA-seq

**Figure 2.**
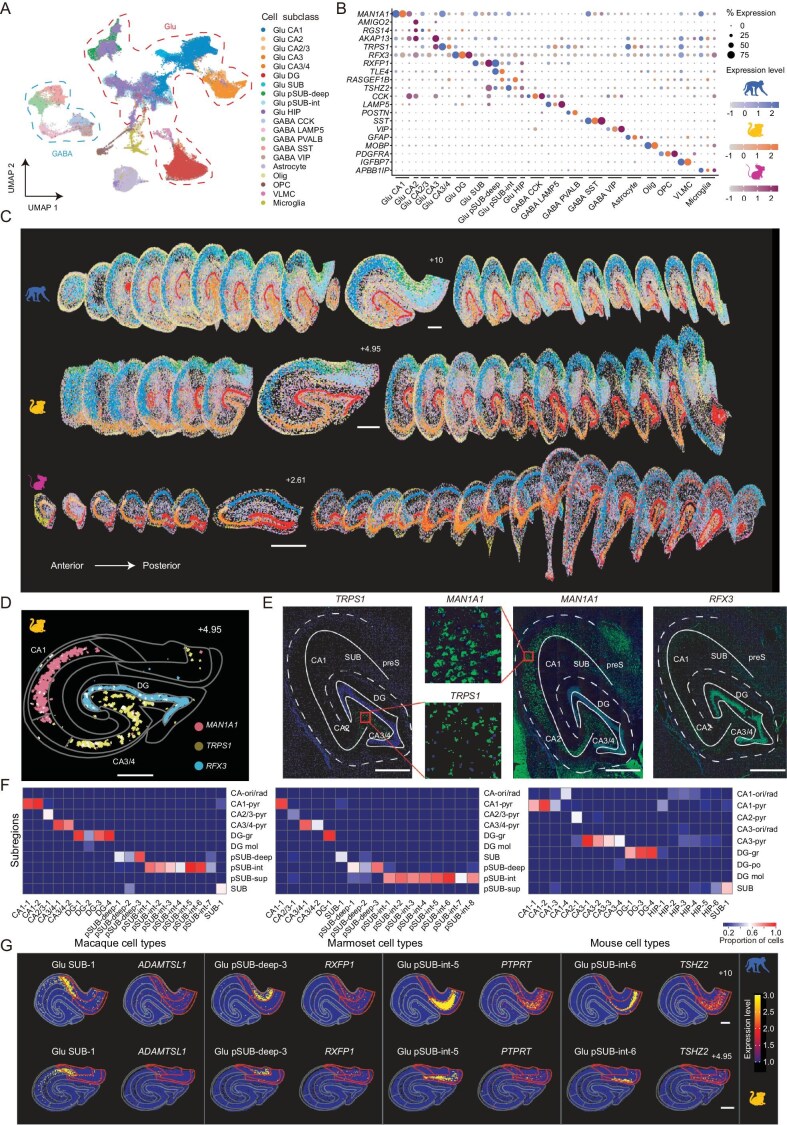
Spatial distribution of subclasses and cell types of hippocampal neurons. (A) The uniform manifold approximation and projection (UMAP) plot of integrated hippocampal cells from macaques, marmosets and mice, with annotated subclasses color-coded. Dashed lines: glutamatergic class (red); GABAergic class (blue). (B) Dot plot displaying marker genes of each subclass of hippocampal cells. The percentage of cells expressing the indicated genes is represented by the dot size, and the expression level of indicated genes by the color intensity (with scales shown in the right). (C) Spatial distribution of various cell types on Stereo-seq maps. Cells on the Stereo-seq maps are color-coded by their subclasses, via registration with snRNA-seq-based subclass annotation, color-coded as in (A). Sections are arranged along the longitudinal axis, with one section shown in the frontal view (EBZ coordinates shown). Scale bars, 1 mm. (D) The expression patterns of example marker genes for CA1, CA3/4 and DG in the marmoset. Contours represent transcriptome-defined subregions. (E) FISH validation of the expression of marker genes shown in (D). Solid lines indicate pyramidal cell layers. Dashed lines indicate boundaries between CA fields and DG and hippocampal structure. (F) Heatmap showing the percentage of cells of each cell type localized in various spatial transcriptome-defined subregions, color-coded with scale shown on the right. (G) Spatial distribution of four subiculum-enriched cell types and the expression profiles of their marker genes, with the expression level color-coded (scale on the right), for macaques (upper panels) and marmosets (lower panels). Contours represent transcriptome-defined subregions, and red contours mark the subiculum complex. Scale bars, 1 mm.

We utilized snRNA-seq analysis to first define hippocampal cell types, and then integrated the snRNA-seq and Stereo-seq data to obtain single-cell spatial transcriptome maps of the macaque, marmoset and mouse hippocampus. Unsupervised clustering analysis using snRNA-seq data of macaque, marmoset and mouse hippocampal cells revealed 10 glutamatergic (Glu) subclasses and 5 GABAergic subclasses of neurons, and 5 subclasses of non-neuronal cells (Fig. 2A). Most subclasses comprised cells from all three species based on standard maker genes (Fig. 2B), whereas Glu CA2 and CA3 subclasses were only identified in mice (Fig. S2A and S2B). Each subclass was then further divided into multiple subclusters (hereafter defined as ‘cell types’, Fig. S2C and S2D; see marker genes in Table S3). Each cell in the spatial transcriptome (Stereo-seq) map was identified by an automatic segmentation method described previously [25] and registered into a cell type based on the highest correlation of its transcriptomic profile with those of snRNA-seq-based cell types (see Materials and Methods). MetaNeighbor analysis showed that transcriptomic profiles were well preserved after registration, and cell types between adjacent sections exhibited overall high similarity (Fig. S2E–S2G). Moreover, cell types were reliably registered onto Stereo-seq maps in biological replicates of the three species (Fig. S3), supporting the reliability of cell types and their spatial registrations across animals.

We found that most glutamatergic cell types exhibited subregion-specific spatial distributions (Fig. 2C), as exemplified by localized spatial distributions of their marker genes such as *MAN1A1* for CA1, *TRPS1* for CA3/4, and *RFX3* for DG in the marmoset hippocampus (Fig. 2D). These subregion-specific distributions were further verified by the FISH analysis of hippocampal sections at similar locations (Fig. 2E and Fig. S3). Therefore, we annotated nine glutamatergic subclasses with ‘Glu’ and the name of their corresponding spatial transcriptome clusters as CA1, CA2/3 and pSUB-deep, and one subclass with ‘Glu-HIP’ due to the absence of subregion specificity. On the other hand, the five GABAergic subclasses were annotated by ‘GABA’ and the name of their typical marker genes such as *SST* and *VIP*, because the majority of these cell types were found to be distributed without clear subregion specificity. Similarly, the five subclasses of non-neuronal cells were annotated as ‘Astrocyte’, ‘Olig’ (Oligodendrocyte), ‘OPC’ (Oligodendrocyte progenitor cell), ‘VLMC’ (vascular and leptomeningeal cell) and ‘Microglia’ based on conventional marker genes for these cell types. For clarity of presentation, we have used these subclass annotations for data shown in Fig. 2A and B.

Within each subclass, various cell types were annotated by the subclass name and additional numbers. For instance, class ‘Glu CA1’ in macaque was further categorized into cell type ‘Glu CA1-1’ and ‘Glu CA1-2’ (Fig. S2A). Quantification of the spatial distribution of various cell types confirmed that the majority of glutamatergic cell types were localized in specific subregions defined by spatial transcriptome profiles described earlier (Fig. 2F). Notably, we identified a large number (>10) of cell types in the ‘pSUB-deep’ and ‘pSUB-int’ subregions (as defined in Fig. 1) in macaques and marmosets, and most of them exhibited clear laminar distribution (Fig. 2G). For example, ‘Glu pSUB-deep-3’ was located mainly in the ‘pSUB-deep’ subregion (marked by *RXFP1*). ‘Glu pSUB-int-5’ was located in the intermediate layer of the pre- and post-subiculum (‘pSUB-int’) and shared the same maker gene *PTPRT*. ‘Glu pSUB-int-6’ (marked by *TSHZ2*) was located in an even thinner lamina within the ‘pSUB-int’ subregion (Fig. 2G). The lamina-specific distribution of subicular cell types indicates the organizational complexity of the subicular complex in primates and may support their distinct physiological functions.

**Figure 3.**
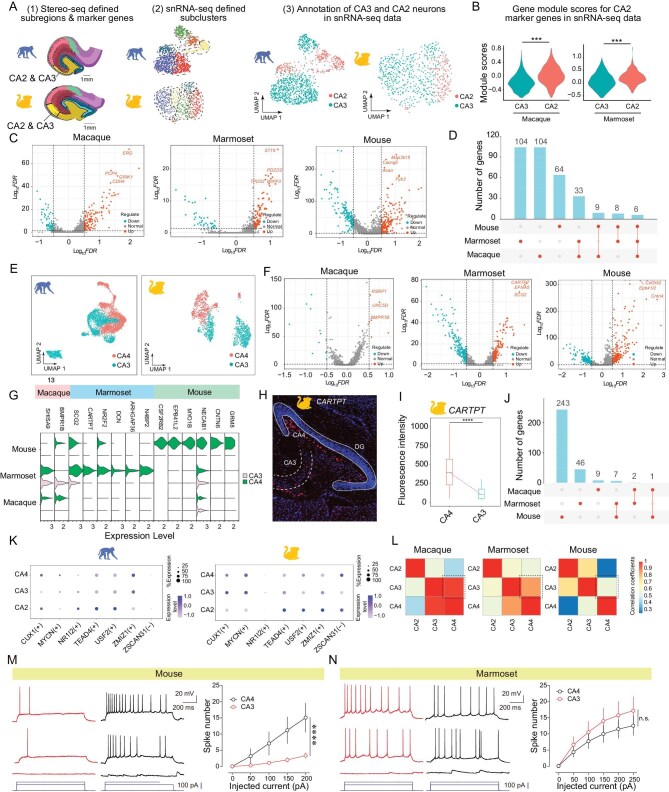
Cross-species comparison of transcriptomic differences among CA2, CA3 and CA4 cells. (A) Procedures to annotate CA2 and CA3 neurons in snRNA-seq data from macaques and marmosets, respectively. (1) Further clustering of CA2/3 Stereo-seq data into CA2 and CA3 pyramidal cells based on the soma location (marked by dashed lines). (2) UMAP showing subclusters of snRNA-seq data of ‘Glu CA2/3’ subclass. (3) Annotating subclusters of snRNA-seq data as CA2 (red) and CA3 (blue) neurons based on marker genes identified in Stereo-seq subclusters. (B) Gene module scores for top 10 marker genes of spatial transcriptome-defined CA2 subregion in snRNA-seq data (CA3 vs. CA2, ****P < 0.0001 for all, unpaired *t*-test). (C) Volcano plots showing DEGs for CA2 and CA3 neurons in macaques, marmosets and mice. (D) Summary of the number of DEGs, either species-specific (first three bars) or shared by two or three species (connected by lines). (E) The classification of CA4 (red) and CA3 (blue) glutamatergic neurons in macaques and marmosets, using the same procedure as in (A). (F) Volcano plots showing DEGs for CA4 and CA3 glutamatergic cell subtypes in the three species. (G) Violin plots showing gene expression levels of representative DEGs enriched in CA4 neurons in all three species. (H) FISH assay of CARTPT (CA4-enriched) expression in the marmoset hippocampus. Solid line, granule cell layer of DG; dashed line, pyramidal cell layer of CA3. (I) Quantification (box plots) of FISH signal intensity of CARTPT expression in CA4 and CA3, expressed as density of field of view (FOV). Circles represented the mean in each group (CA3 vs. CA4, ****P < 0.0001, unpaired *t*-test). (J) Summary of the number of DEGs for CA3 and CA4, either species-specific (first three bars) or shared by two or three species (connected by lines). Note the reduced numbers of DEGs in primates. (K) Dot plots showing the expression pattern of indicated regulons (transcription factors) across CA4, CA3 and CA2 glutamatergic cells in macaques (left) and marmosets (right). The size of the dot represents the percentage of the cells expressing indicated genes, and the color intensity of the dot indicates the expression level. (L) Heatmaps showing the correlation coefficients (CCs) of gene expression profiles among CA4, CA3 and CA2 glutamatergic cells in three species. The CC value is color-coded with the scale shown on the right. Note that the similarity of the expression profiles between CA3 and CA4 (dashed boxes) in macaques and marmosets is higher than that in mice. (M) Left, example recordings of two pyramidal cells in mouse CA3 (red) and CA4 (black). Right, summary of spike numbers evoked by current injections. CA3: *n* = 11 cells from five animals. CA4: *n* = 12 cells from five animals. Two-way ANOVA, ****P < 0.0001. (N) Left, example recordings of two pyramidal cells in marmoset CA3 (red) and CA4 (black) in response to three steps of depolarizing currents (depicted below). Scale bars, 20 mV and 200 ms. Right, summary of spike numbers (mean ± SEM) evoked by various current injections. CA3: *n* = 18 cells from three animals. CA4: *n* = 16 cells from three animals. Two-way ANOVA, *P* = 0.65.

We found that the mouse CA2 and CA3 harbored distinct spatial transcriptomic profiles (‘CA2-pyr’ and ‘CA3-pyr’), whereas CA2 and CA3 shared a similar spatial transcriptomic profile (‘CA2/3-pyr’) in marmosets and macaques (Fig. 1C). To further identify differentially expressed genes (DEGs) between CA2 and CA3 in primates, we performed further clustering analysis within the spatial transcriptome profile of ‘CA2/3-pyr’ and then used their DEGs to annotate subclusters of the ‘Glu CA2/3’ subclass in the snRNA-Seq data of glutamatergic neurons in the CA2 and CA3 of marmosets and macaques (Fig. 3A). The top 10 DEGs defined by the spatial transcriptome robustly showed different gene module scores in snRNA-seq data (Fig. 3B; see examples in Fig. S4A and see Stereo-seq DEG list in Table S4). Consequently, many more DEGs between CA2 and CA3 were identified in snRNA-seq data (Fig. 3B), which were mostly distinct among three species (Fig. 3C and D; see snRNA-seq DEG list in Table S4).

Using similar approaches, we identified DEGs for CA3 and CA4 neurons in primates by further clustering analysis of the spatial transcriptomic profile of ‘CA3/4-pyr’ (Fig. 3E). Some DEGs such as *EPHA6* and *CARTPT* were reliably identified in the CA4 subregion of macaques and marmosets, respectively (Fig. S4B). Furthermore, more DEGs were identified from the snRNA-seq data of macaques (e.g. *RSRP1, UNC5D* and *BMPR1B*) and marmosets (e.g. *SCG2, CARTPT* and *EFNA5*) (Fig. 3E and F; see DEG list in Table S4). We validated the differential CA3 vs. CA4 expression by the quantitative FISH assay for the expression of a neuropeptide-encoding gene *CARTPT*, which was preferentially expressed in marmoset CA4 (Fig. 3G–I). Notably, the number of DEGs in CA3 and CA4 progressively decreased from mice to marmosets and to macaques (Fig. 3J). In line with this finding, CA3 and CA4 neurons in primates exhibited a similar gene expression pattern of transcription factors, neurotransmitter receptors and ion channels (Fig. 3K and Fig. S4C and S4D), and the transcriptomic similarity (expressed as correlation coefficients of the 100 most variable genes) between CA3 and CA4 neurons increased from mice to marmoset and to macaques (mouse ‘CA4’ was also referred to as the hilus and corresponded to the transcriptome-based subregion ‘DG-po’, Fig. 3L). In contrast, there were no such gene expression similarities between CA2 and CA3 neurons in primates (Fig. 3L and Fig. S4C and S4D).

The above results on ion channels in CA3 and CA4 suggest that the difference in neuronal excitability of CA3 and CA4 neurons is smaller in mice than in primates. To test this possibility, we performed whole-cell patch-clamp recording in acute brain slices of marmosets and mice to measure evoked spike numbers as a function of injected current amplitudes (intrinsic excitability expressed as an I–V curve). Consistently, we found that I–V curves and spike thresholds for mouse CA3 and CA4 neurons were significantly different (Fig. 3M and Fig. S4G), whereas those of marmoset CA3 and CA4 neurons were not (Fig. 3N and Fig. S4H). The difference in I–V curves and spike thresholds between mouse CA3 and CA4 neurons may have to do with ion channel expressions such as Na^+^ channel *SCN* subunits (Fig. S4F). Taken together, these results suggest an evolutionary reduction of transcriptomic differences of glutamatergic cell types in CA3 and CA4, suggesting a progressive functional convergence of these two subregions in primates.

### Cross-species analysis of GABAergic and glial cell types

**Figure 4.**
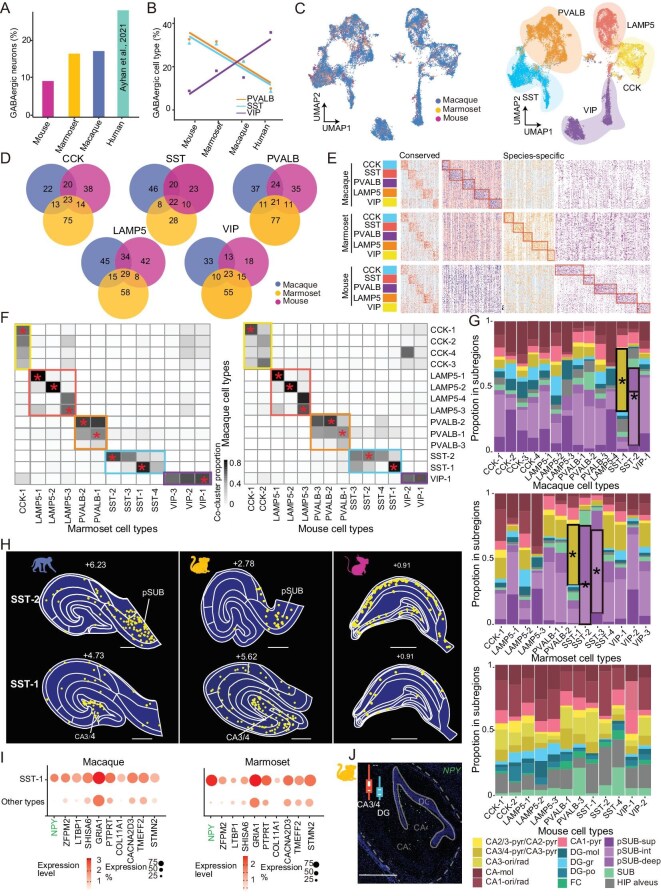
Cross-species comparison of GABAergic cell types. (A) The percentage of GABAergic cells among all neurons in mouse, marmoset, macaque and human hippocampus. (B) The percentage of three subclasses of GABAergic cells (PVALB, SST and VIP) among all GABAergic cells in the hippocampus of four species. (C) UMAP clustering of GABAergic cells from the three species. Cells are color-coded by the species (left) and by subclasses (right). (D) Venn diagrams showing the number of shared and distinct GABAergic marker genes among three species. (E) Heatmap showing the conserved and species-specific marker genes in each subclass of GABAergic cells in macaques, marmosets and mice. (F) Cross-species comparisons of GABAergic cell types between macaques and marmosets (left), and between macaques and mice (right). The gray level indicates the co-clustering proportion of cells that belong to the same cell type in both species. Color boxes indicate GABAergic subclasses. Note that all subclasses are largely conserved but the cell type diversity within the subclasses is species-dependent. (G) Stacked bar plots showing the percentages of neurons in spatial transcriptome-defined subregions (color-coded, legend shown below) for each GABAergic cell type in all three species. The subregion with high dominance (proportion > 0.4) is marked with an asterisk. (H) Spatial distribution of GABA SST-1 (lower) and SST-2 (upper) cell types in the hippocampal sections at comparable longitudinal locations in all three species. (I) Dot plots showing the top expressed genes in the GABA SST-1 cell type. The *NPY* gene was highly enriched in this cell type, compared to other GABA cell types. Dot size, percentage of cells expressing the indicated gene; dot color intensity, expression level. (J) FISH assay showing enriched expression of *NPY* in the CA4 region of marmoset hippocampus. Scale bars, 1 mm.

GABAergic neurons are key regulators for neuronal activity in local circuits. To understand the evolutionary changes of GABAergic cells, we have analyzed hippocampal GABAergic cells in the snRNA-seq data of macaques, marmosets and mice, together with previously reported human snRNA-seq data [22]. We found that the percentage of GABAergic cells among all sampled hippocampal cells progressively increased in the sequence of evolutionary order, with the highest percentage in humans (Fig. 4A and Fig. S5A). A similar trend was also reported for GABAergic neurons in the motor cortex [30].

Our clustering analysis based on snRNA-seq data of macaques, marmosets and mice yielded five GABAergic subclasses expressing marker genes *PV, CCK, VIP, SST* and *LAMP5*, respectively (see Fig. 2A), similar to that found in humans [23]. Further analysis showed that the percentage of each GABAergic subclass among all GABAergic cells was markedly different across species (Fig. 4B). Notably, the percentage of VIP GABAergic cells, which are known to be highly involved in disinhibitory local circuits [36], was the highest in humans and lowest in mice. In contrast, the percentages of PV and SST cells showed the opposite trend (Fig. 4B). This suggests quantitative changes in the relative proportion of three major GABAergic subclasses during evolution from rodents to primates.

We next compared the gene expression profiles of these GABAergic subclasses among the mouse, marmoset and macaque. Overall, the number of shared marker genes was the highest between macaque and marmoset for all five GABAergic subclasses (Fig. 4C–E), although most marker genes exhibited enriched expression in only one species (Fig. 4E). Further pairwise comparison of cell types across species showed that GABAergic cell types exhibited largely similar gene expression patterns, as reflected by high percentages of co-clustered cells in the integrative clustering analysis (Fig. 4F). We found that most GABAergic cell types were present in various hippocampal subregions, but their percentages differed among the three species (Fig. 4G). Moreover, we examined the spatial distribution of individual GABAergic cell types. Only in primates, we observed localized distribution of the ‘GABA SST-1’ cell type in the ‘CA3/4-pyr’ subregion and the ‘GABA SST-2’ cell type in the ‘pSUB’ subregion (Fig. 4G; see details in Table S5). The preferential distribution of the ‘SST-1’ cell type was reliably observed across sections and exemplified by the *NPY* expression (Fig. S5C–S5E), which was expressed much more highly in the ‘GABA SST-1’ cell type than in all other GABAergic cell types (Fig. 4I). This was further validated by the FISH assay in the marmoset hippocampal sections (Fig. 4J). These results indicate that although GABAergic cell types are all present in the three species, there were evolutionary changes from rodents to primates in their relative proportion and spatial distribution. Finally, we measured the spatial distribution of glial cell types among transcriptome-defined subregions, and found that some subregion-enriched cell types in marmosets and mice, as exemplified by the ‘astrocyte-1’ cell type, were enriched in the ‘CA-mol’ subregion of mice (Fig. S6).

### Cross-species analysis of glutamatergic cell subtypes

**Figure 5.**
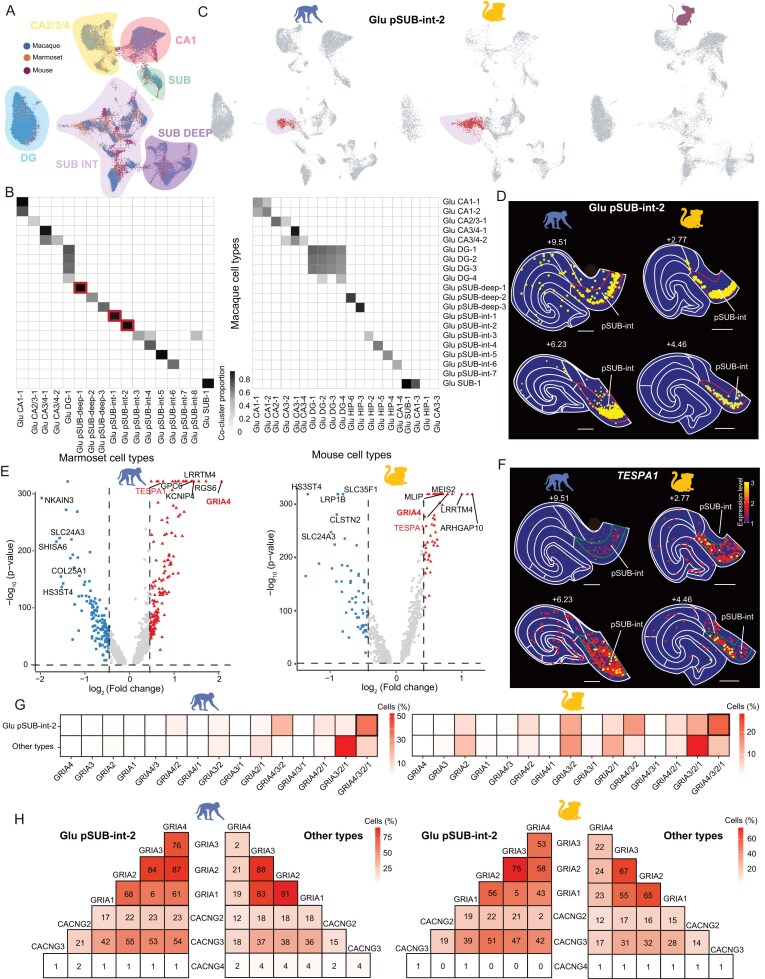
Cross-species comparison of glutamatergic cell types. (A) UMAP visualization of integrated snRNA-seq data for pooled glutamatergic neurons from macaque, marmoset and mouse hippocampus. (B) Cross-species comparisons of hippocampal glutamatergic cell types between macaques and marmosets (left), and between macaque and mouse (right). The gray level indicates the proportion of co-clustered cells that belong to the same cell type in both species. (C) UMAP data of individual species extracted from that shown in (A). Red dots, glutamatergic cells of the ‘Glu pSUB-int-2’ cell type, which is found only in macaques and marmosets. (D) Spatial distribution of the ‘Glu pSUB-int-2’ cell type in two sections (EBZ coordinates shown above) of the macaque and marmoset hippocampus. The pSUB-int subregion is outlined by red lines. Scale bars, 1 mm. (E) Volcano plots showing DEGs for the ‘Glu pSUB-int-2’ cell type and other glutamatergic cell subtypes in macaques (left) and marmosets (right). (F) The expression pattern of the marker gene *TESPA1* for the ‘Glu pSUB-int-2’ cell type; same sections as in (D), with expression level color-coded by the scale bar at the right. The pSUB-int subregion is outlined by green lines. Scale bars, 1 mm. (G) Heatmap showing percentages of cells expressing various AMPA receptor subunit genes for the ‘Glu pSUB-int-2’ cell type and the rest of the glutamatergic cell types in macaques (left) and marmosets (right). (H) Heatmap showing percentages of cells co-expressing AMPA receptor subunit genes and three stargazin genes in the ‘Glu pSUB-int-2’ cell type and the rest of the glutamatergic cell types in macaques (left) and marmosets (right), respectively. The number in the box depicts the percentage, with color bars shown on the right. Scale bars, 1 mm.

We further investigated whether there are primate-specific glutamatergic cell types in the three species, using consensus clustering followed by co-clustering matrix analysis (Fig. 5A; see details in Table S6). Notably, three glutamatergic cell types (pSUB-deep-1, pSUB-int-1 and pSUB-int-2) were present in both macaques and marmosets, but not in mice (Fig. 5B and C, and Figs S7A and S8A), indicating the evolutionary emergence of primate-specific cell types in the subicular complex. Further integrative analyses with published mouse datasets [37] and human datasets [38,39] validated the primate specificity of these three glutamatergic cell types (Figs S9 and S10). Interestingly, the ‘Glu pSUB-int-2’ cell type exhibited a highly localized distribution across sections and animals, and expressed marker genes such as *TESPA1* (Fig. 5D and Fig. S7B). Compared to other types of subicular neurons, Glu pSUB-int-2, Glu pSUB-int-1 and Glu pSUB-deep-1 neurons in macaques and marmosets shared preferential gene expressions such as *GRIA4, PTPRK* and *KIAA1217*, respectively (Fig. 5E and Fig. S7C–S7E). Remarkably, all three primate-specific cell types exhibited prominent co-expression of marker genes enriched in macaque cortical layers such as layer 2/3 and layer 5/6 (Fig. S7F and S7G), suggesting a molecular and cellular basis for cortical-like functions in the primate subicular complex. Furthermore, the Gene Ontology (GO) analysis revealed stronger scores for pathways related to synaptic transmission and major depression disorder (MDD) [40], for which GluR4 was suggested to be a potential diagnostic biomarker [41] (Fig. S7H and S7I).

Given the preferential expression of the AMPA receptor subunit gene *GRIA4* in the ‘Glu pSUB-int-2’ cell type, we analyzed the expression profiles of all combinations of AMPA receptor subunits in the subicular complex of macaques and marmosets. We found that ‘Glu pSUB-int-2’ preferentially expressed subunits *GRIA1/2/3/4*, whereas other cell types including ‘Glu pSUB-deep-1’ and ‘Glu pSUB-int-1’ preferentially expressed subunits *GRIA1/2/3* (Fig. 5G and Fig. S7J). The AMPA subunit genes in ‘Glu pSUB-int-2’ cell types generally showed higher co-expression probability with the AMPA receptor auxiliary subunit *CACNG3* than those in other cell types (Fig. 5H). Taken together, our results showed that the primate-specific glutamatergic cell types exhibit laminar preferences in the subicular complex, and this laminar organization may contribute to different hippocampal functions by differential expression of specific sets of neurotransmitter receptor subunits.

### Heterogeneous distribution of glutamatergic neurons along hippocampal axes

**Figure 6.**
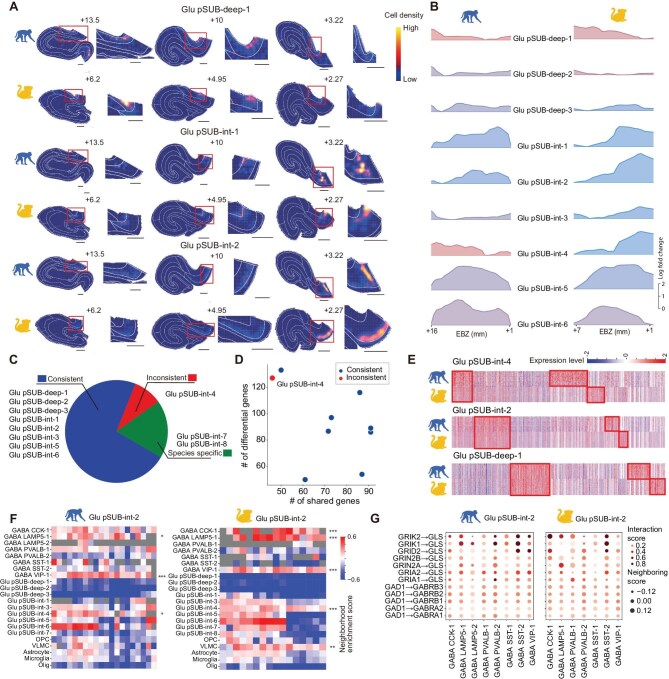
Longitudinal heterogeneity in the distribution of glutamatergic cell types. (A) Spatial distributions (color-coded cell density, scale bar at right) of primate-specific subicular glutamatergic cell types in three representative sections at anterior, intermediate and posterior EBZ coordinates respectively (shown above) along the longitudinal axis of macaque and marmoset hippocampus. The red box area in each section is enlarged and shown on the right. Scale bars, 1 mm. (B) Spatial distribution of all subicular cell types along the longitudinal axis in macaques (upper) and marmosets (lower). Left, heterogeneity (expressed as log of the standard variation) of cell densities along the longitudinal axis for each cell subtype. The log of cell number for each cell type is color-coded with the color bar shown above each plot. Right, ridge plot showing relative cell densities along the longitudinal axis. (C) Pie chart illustrating the consistency of spatial distribution patterns of various subicular glutamatergic cell types along the longitudinal axis between macaques and marmosets. (D) Comparison of overlapping and species-specific marker genes of subiculum glutamatergic cell subtypes between macaques and marmosets. Cell types with consistent and inconsistent longitudinal distribution are shown in blue and red, respectively. (E) Heatmap showing the expression level of conserved (left boxes) and species-specific (right boxes) marker genes for Glu pSUB-deep-1, pSUB-int-2 and pSUB-int-4 between macaques and marmosets. (F) Heatmap depicting neighborhood enrichment score of Glu pSUB-int-2 with various cell types along the anterior–posterior axis of the macaque and marmoset hippocampus. The score is color-coded with the scale bar shown on the right. Gray color indicates that the score was not computable due to a low cell number of paired cell types in that hippocampal section. Note that the interaction between Glu pSUB-int-2 and GABA LAMP5-1 and VIP-1 was significantly stronger in the posterior part of the anterior–posterior axis. Spearman correlation test: *P < 0.05, **P < 0.01, ***P < 0.001. (G) Dot plots showing the ligand–receptor integration strength between Glu pSUB-int-2 and different GABAergic cell subtypes in macaques and marmosets. Dot size indicates the neighborhood enrichment score of GABA neurons, and dot color represents interaction score for each pair of ligands and receptors.

The ventral and dorsal parts of the rodent hippocampus (homologs of anterior and posterior portions of the primate hippocampus) are known to exhibit distinct brain-wide connectivity, gene expressions and functions along the longitudinal axis, as well as the proximal–distal axis and superficial to deep layers [2,15,17,18,42–49]. In this study, we first systematically mapped the distribution of all hippocampal cell types in all three species along the longitudinal axis, and then focused on primate subicular cell types, which exhibited a high diversity and distinct laminar distributions. Our results showed that whereas the longitudinal distribution of GABAergic and non-neuronal cell types along the longitudinal axis was largely uniform (expressed as the longitudinal heterogeneity; Fig. S11A), nearly all transcriptome-defined glutamatergic cell types exhibited larger longitudinal heterogeneity (Fig. 6A and B), which was reliably observed in animal replicates (Fig. S11A and S11B). Moreover, cross-species comparison showed that the same glutamatergic cell type exhibited similar preferential distribution in both the macaque and marmoset hippocampus, as exemplified by the preferentially anterior distribution of ‘Glu CA3/4–2’ and posterior distribution of ‘Glu pSUB-int-2’ cell types, respectively (Fig. 6B and Fig. S11A). Distribution analysis from superficial to deep layers showed that more than half of subicular cell types in the primate subicular complex exhibited clear laminar distribution (Fig. S12A). Of note, the ‘Glu CA3-2’ cell type in mice showed preferential distributions enriched in the distal and deep parts of the *str. pyramidale* (Fig. S12A). Further analysis revealed many genes in CA1 showing specific preference along the proximal–distal axis and from superficial to deep layers (Fig. S12B; see details in Table S7), as exemplified by *CCK* enriched in the superficial layer and *SCG3* enriched in the proximal part of the *str. pyramidale* of macaque CA1 (Fig. S12C). These gene markers may provide molecular handles to study the functional heterogeneity along multiple hippocampal axes in primates and rodents.

The subicular subregions showed the highest cell diversity in both macaques and marmosets (Fig. 2F), and many cell types such as ‘Glu pSUB-int-6’ and ‘Glu pSUB-deep-3’ cell types exhibited laminar distributions along the superficial–deep axis of the subicular complex. For the three primate-specific subicular cell types described in Fig. 5, ‘Glu pSUB-deep-1’ was predominantly found in the anterior hippocampus, whereas ‘Glu pSUB-int-1’ and ‘Glu pSUB-int-2’ were enriched in the posterior hippocampus (Fig. 6A; see animal replicates in Fig. S11B). Furthermore, many other glutamatergic cell types located in the ‘pSUB’ subregion showed preferential distribution along the longitudinal axis (Fig. 6B), and many of their preferences were similar (Fig. S11C). We categorized cell types as anterior-enriched, posterior-enriched or uniformly distributed in each species and found that most of the subicular cell types had the same preferences between the two primate species except for ‘Glu pSUB-int-4’, which showed opposite distribution patterns between species (Fig. 6B and C). Comparison of the top 200 marker genes for each cell type between macaques and marmosets showed little overlap of marker genes between these two oppositely distributed cell types (Fig. 6D; see marker genes in Table S8). In contrast, cell types with similar distribution patterns in the two species shared a much higher number of conserved marker genes (Fig. 6D and E). Furthermore, we conducted enrichment analysis based on the shared and differential genes between species using Synaptic Gene Ontologies. We found that these shared genes were enriched in synapse-related terms, indicating conserved neuronal functions (Fig. S11D and S11E). On the other hand, species-specific marker genes were enriched in GO terms including cell morphology and cell–cell adhesion, implicating differential cell–cell interactions and morphogenesis between the two species (Fig. S11E).

Previous findings have revealed that cells with close proximity had higher probabilities of forming synaptic connections [29,30]. Given the preferential distribution of various glutamatergic cell types along the longitudinal axis, we next examined the neighborhood of each glutamatergic cell type by computing the spatial neighborhood enrichment score (defined by the number of cells within a given distance) for various types of glutamatergic, GABAergic and non-neuronal cells. We found that, for the neuron ‘Glu pSUB-int-2’ cell type, it showed overall high neighborhood scores with GABAergic cell types in the posterior hippocampus, whereas it exhibited high neighborhood scores with the ‘Glu pSUB-int-6’ cell type only in the anterior hippocampus (Fig. 6F). For the ‘Glu pSUB-deep-1’ cell type, it exhibited high neighborhood scores with non-neuronal cells only in the posterior hippocampus (Fig. S11F). Finally, we examined the relationship between GABAergic cell types in the neighborhood of ‘Glu pSUB-int-2’ and their GABAergic receptor subunit expressions from the snRNA-seq data. We found that the G protein-gated inwardly rectifying potassium (GIRK) channel subunit gene *GRIK2* was enriched in the neighborhood with many GABAergic cell types such as ‘GABA SST-2’ (Fig. 6G and Fig. S11G). Thus, the distinct preferential distribution of various cell types along the anterior–posterior axis and the composition of cell types preferentially localized in their neighborhood provided the basis for local cellular interaction underlying functional heterogeneity along the longitudinal axis of the primate hippocampus.

### Longitudinal heterogeneity in electrophysiological properties of CA1 neurons

**Figure 7.**
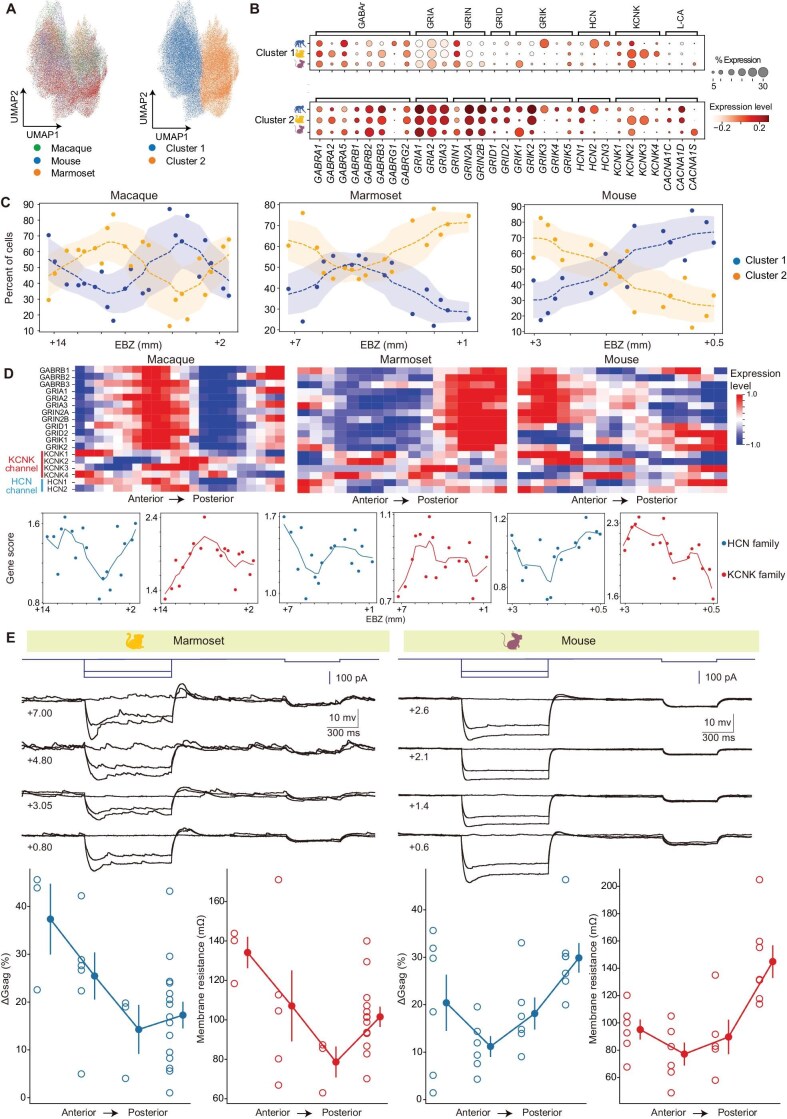
Heterogeneity of physiological properties of neurons along the longitudinal axis. (A) UMAP embeddings of CA1 single-cell spatial transcriptome data from macaques, marmosets and mice. Cells are colored by species (left) and unsupervised clusters (right). (B) Dot plots showing the expression patterns of glutamate receptors, GABA receptors and ion channel genes between clusters 1 and 2 in macaques, marmosets and mice. (C) Spatial distribution of the two CA1 cell clusters along the longitudinal axis in three species. (D) Upper panels, heatmap showing the expression pattern of marker genes for cluster 2 in sections ordered along the longitudinal axis in three species. Gene expression is calculated by aggregating all counts of CA1 neurons on the chip and then z-scored along the longitudinal axis. Lower panels, gene scores for two families of potassium channels, HCN (blue) and KCNK (red), were computed as a sum of gene subunit expressions and plotted as a function of EBZ coordinates of CA1 neurons (lower panels). (E) Slice recording of CA1 neurons in various longitudinal locations from the marmoset (left column) and mouse (right column) hippocampus. Upper, example recordings of pyramidal cells at four longitudinal locations (EBZ coordinates shown on the left) of the marmoset and mouse CA1, respectively. Lower, ∆Gsag (blue) and membrane resistance (red) as a function of the longitudinal locations of recorded cells (ordered from anterior to posterior). One-way ANOVA, *P* < 0.05 for all.

The CA1 pyramidal neurons are the main output of the tri-synaptic core circuit (DG–CA3–CA1) of the hippocampus. The heterogeneity in electrophysiological properties of CA1 neurons along the longitudinal axis may underlie differences in connectivity and functional properties [2,50]. Thus, we performed clustering analysis of snRNA-seq data on the expression profiles of ion channels and transmitter receptors, which are critical for physiological properties of a neuron. Two clusters of CA1 neurons in each species showed preferential expression of specific sets of genes for glutamate and GABA receptors, as well as for ion channels such as *CACNA1C* and *HCN1* (Fig. 7A and B; see marker genes for the two groups in Table S9). Interestingly, CA1 neurons in the two groups exhibited distinct patterns of distribution along the longitudinal axis (Fig. 7C), suggesting co-localized longitudinal distributions for transmitter receptors and ion channels.

Indeed, we found that the averaged gene expression of various transmitter receptors and ion channels for all CA1 neurons exhibited localized distributions along the longitudinal axis. Among these genes, most primate genes exhibited much higher expression in the anterior and posterior parts than in the intermediate part of the CA1 subregion (see cluster 2 marker genes *GABRB1, GRIA1* and *HCN1*; Fig. 7D), whereas only a few mouse genes showed the similar trend (exemplified by *HCN1* and *HCN2*; Fig. 7D). As the hyperpolarization-activated cyclic nucleotide-gated (HCN) channel mediates the hyperpolarization-activated current (I_h_), we performed whole-cell patch-clamp recording in acute brain slices of marmosets and mice, activated HCN channels by hyperpolarization and recorded changes in membrane conductance (∆G_sag_) as a proxy for I_h_ and the expression of HCN channels [51]. We recorded CA1 neurons from brain slices at four different longitudinal locations, and found that ∆G_sag_ values were larger in the anterior and posterior sections than those in the intermediate sections from marmosets (Fig. 7E). Similar heterogeneity of ∆G_sag_ was also found in ventral-to-dorsal hippocampal sections in mice (homologs of anterior-to-posterior sections in primates; Fig. 7E). The membrane resistance, which is negatively correlated with ion channel expression in general, showed similar heterogeneity of ∆G_sag_ for neurons recorded along the longitudinal axis (Fig. 7E). Indeed, this was negatively correlated with the gene scores of *KCNK* channel subunits (Fig. 7D, red lines in lower panels). These results revealed non-linear heterogeneous expression of transmitter receptors and ion channels along the longitudinal axis that may provide the cellular basis for physiological heterogeneity. Taken together, these results suggest that CA1 neurons in primates and mice share some evolutionarily conserved longitudinal heterogeneity of intrinsic neurophysiological properties.

## DISCUSSION

By integrating spatial transcriptomic and snRNA-seq data for the macaque, marmoset and mouse hippocampus, we have systematically characterized the gene expression profiles of hippocampal subregions and identified diverse cell types, as well as their spatial distribution patterns in the hippocampus. Our study revealed spatial transcriptome-based hippocampal subregions and primate- and lamina-specific glutamatergic cell types in the subicular complex and validated these findings in our human spatial transcriptome analysis. We also identified evolutionary changes in the composition of various GABAergic cell types, as well as their subregion-specific distributions. Interestingly, gene expression profiles showed a distinct difference in mouse CA3 and CA4 subregions, but such a difference became progressively diminished in marmosets and macaques, as exemplified by the similarity in the intrinsic neuronal excitability between CA3 and CA4 neurons in primates. We also found that the profiles of distribution along the longitudinal axis were in general highly heterogeneous for glutamatergic cell types but not for non-neuronal cell types. Furthermore, the longitudinal profiles of subunit gene expression for ion channels and neurotransmitter receptors were also highly heterogeneous, and corresponding functional consequence was further demonstrated by variation of HCN channel currents of CA1 neurons along the longitudinal axis. Our study yielded gene expression and cell type atlases of the hippocampus for three mammalian species, providing a comprehensive resource for studying the molecular and cellular basis underlying the evolution and function of the hippocampus. These data can be accessed online (https://digital-brain.cn/cross-species/hipp/).

### Molecular anatomy for hippocampal subregions across species

Our study showed that the spatial transcriptome analysis was a useful approach for defining hippocampal subregions based on molecular features. We found that these subregions were stable in various Stereo-seq sections along the longitudinal axis and similar among the three species. Some subregion marker genes were consistent with previous findings obtained by bulk RNA sequencing [19]. Notably, our spatial transcriptomic mapping revealed that the gene expression pattern in the *str. radiatum* was similar to that in the *str. oriens* but distinct from that in the *str. lacunosum-moleculare*. This suggests the existence of distinct transcripts in distal vs. apical/basal dendritic domains that may be linked to the nature of projecting axons (perforant pathway vs. Shaffer collaterals). We also found that, whereas the transcriptomic profile of CA2 remained distinct from that of CA3 and CA1, there was an increasing similarity in transcriptomic profiles and neurophysiological properties of CA3 and CA4 neurons from mice to marmosets and macaques, implicating convergence of functions of these two areas in primates. This is surprising, since primate evolution is expected to yield more diverse subregions due to cortical expansion and more complex brain functions. The non-canonical mossy cell axons recently identified in mouse CA4 (hilar region of DG) that exhibited similar projections as CA3 neurons [16] could be a prelude to the evolutionary convergence of CA3 and CA4 in primates.

The finding of laminae structures in the primate subicular complex by spatial transcriptome mapping suggests that the organization principle of the primate subicular complex may be similar to that of the cerebral cortex, with neurons in different layers responsible for distinct connectivity and physiological functions. This is further supported by our finding that many glutamatergic cell types in primates showed subicular lamina-specific localization, and some of them exhibited gene expression patterns similar to those in cortical layer 2/3 or layer 5/6 neurons. Presubicular cells could exhibit lamina-specific cell morphologies and intrinsic properties [52,53], yet, whether subicular lamina-specific cell types exhibit distinct neuronal connectivity for implementing their physiological functions remains to be investigated. Notably, all three primate-specific glutamatergic cell types were found to be localized in the pSUB subregion. Among them, the ‘Glu pSUB-int-2’ cell type is of particular interest because of its high expression of the AMPA receptor subunit gene *GRIA4* that could result in larger synaptic currents for synaptic transmission and plasticity [54].

### Primate-specific compositions of GABAergic cell types

Cross-species comparison among four mammalian species (mouse, marmoset, macaque and human) revealed a progressive elevation in the abundance of hippocampal GABAergic neurons from mice to humans, similar to that found in cross-species studies of the cerebral cortex [14,55]. Moreover, the composition of GABAergic subclasses in primates differed substantially from that in mice, as reflected by the higher abundance of VIP cells than of SST and PV cells. The presence of more GABAergic VIP cells, which are known to mediate disinhibition via innervating other types of interneurons [36] and could send out long-range projections [56], suggests more complex regulation of hippocampal pyramidal cell activity in primates.

All GABAergic cell types in mice showed generally uniform distribution across hippocampal subregions. In contrast, GABA SST-1 and SST-2 cell types in both marmosets and macaques exhibited preferential localization in CA3/4 and pSUB-int subregions, respectively. This suggests that spatial reorganization of SST cells in primates is important for specific physiological functions of the primate hippocampus. We found that SST-1 cells also highly expressed the *NPY* gene. Whether its neuropeptide Y co-released with GABA plays a functional role remains to be determined.

### Heterogeneous distributions of cell types and gene expressions along hippocampal axes

Our study demonstrated that distinct genes in the CA fields, as well as cell types in the subicular complex, exhibited heterogeneous distributions along the proximal–distal axis and superficial to deep layers. These results provide molecular markers for future studies to investigate the functional heterogeneity of CA and subicular neurons, particularly in primates. We also found that hippocampal glutamatergic cell types in general exhibit heterogeneity in their distribution along the longitudinal axis, consistent with diverse functional specializations along the hippocampal longitudinal axis. Our finding of distinct longitudinal profiles of various glutamatergic cell types indicates that such heterogeneity was not due to random variation in cell sampling. This is supported by the finding that non-neuronal cells did not show longitudinal preferences. Although within-cell-type heterogeneity [16,18] in connectivity and function remains to be clarified, the notion that distinct longitudinal profiles of glutamatergic cell types are reliable is further supported by cross-species analysis for similar cell types in the macaque and marmoset subicular complex, which exhibited largely similar longitudinal distributions. This finding suggests that longitudinal functional specializations of various subicular cell types are conserved between macaques and marmosets. The longitudinal profiles of glutamatergic cell types could be linked to the longitudinal heterogeneity of subunit gene expression profiles of ion channels and transmitter receptors. In particular, we examined the longitudinal distribution in the expression of *I*_h_ channel subunit genes *HCN1* and *HCN2*, which showed a roughly ‘U-shape’ longitudinal profile. The functional relevance of such a distribution profile was further supported by the result of our electrophysiological recordings in marmoset and mouse hippocampal slices, which showed a very similar ‘U-shape’ longitudinal distribution of ∆G_sag_ in CA1 neurons in light of the reversed homologous correspondence between marmoset and mouse longitudinal axes of the hippocampus.

In conclusion, we have obtained gene expression and cell type atlases for macaque, marmoset and mouse hippocampus, based on single-cell spatial transcriptomic data. Cross-species comparison revealed primate-specific cell types, their cross-sectional subregion localization and primate-specific subicular laminar distribution, as well as their preferential distribution along the longitudinal axis. The spatial transcriptome-defined subregions and cell types provide an important molecular and cellular basis for future studies of the organization of hippocampus structure, cell type-specific connectomes and physiological function, and evolutionary changes in the mammalian hippocampus.

## MATERIALS AND METHODS

### Animals

All animal procedures (ION-2019011, CEBSIT-2021038, CEBSIT-2021039, NA-047-2020) were performed in accordance with institutional guidelines and were approved by the Institutional Animal Care and Use Committee (IACUC) of the Institute of Neuroscience, CEBSIT, Chinese Academy of Sciences.

### Human tissues

All donors provided informed consent for brain autopsy and the use of their tissue and clinical data for research purposes in compliance with Dutch national ethics guidelines. Additional ethical screening and approval for using post-mortem human brain tissue for molecular profiling was provided by the regional ethical committee in EPN, Stockholm, Sweden (2013/474-31/2).

The detailed materials and methods are available in the Supplementary data.

## Supplementary Material

nwaf595_Supplemental_Files

## Data Availability

The pre-processed data ready for exploration can be accessed and downloaded via https://digital-brain.cn/cross-species/hipp/. All raw data have been deposited in the CNGB Nucleotide Sequence Archive (https://db.cngb.org/search/project/CNP0003026) and are publicly available as of the date of publication. All data were analyzed with standard programs and packages. The codes were freely accessible from https://github.com/tyfei0216/HIP. Additional information required to reanalyze the data reported in this paper is available from the lead contact upon request.

## References

[1] Andersen P, Morris R, Amaral DG et al. The Hippocampus Book. New York: Oxford University Press, 2009.

[2] Strange BA, Witter MP, Lein ES et al. Functional organization of the hippocampal longitudinal axis. Nat Rev Neurosci 2014; 15: 655–69. 10.1038/nrn3785 25234264

[3] Scoville WB, Milner B. Loss of recent memory after bilateral hippocampal lesions. J Neurol Neurosurg Psychiatry 1957; 20: 11–21. 10.1136/jnnp.20.1.11 13406589 PMC497229

[4] O’Keefe J, Nadel L. The Hippocampus as a Cognitive Map. Oxford: Oxford University Press, 1978.

[5] Henke PG . Hippocampal pathway to the amygdala and stress ulcer development. Brain Res Bull 1990; 25: 691–5. 10.1016/0361-9230(90)90044-Z 2289157

[6] Tottenham N, Sheridan MA. A review of adversity, the amygdala and the hippocampus: a consideration of developmental timing. Front Hum Neurosci 2009; 3: 68. 10.3389/neuro.09.068.2009 20161700 PMC2813726

[7] Andersen P, Bliss TV, Skrede KK. Lamellar organization of hippocampal pathways. Exp Brain Res 1971; 13: 222–38. 10.1007/BF00234087 5570425

[8] Aggleton JP, Christiansen K. The subiculum: the heart of the extended hippocampal system. Prog Brain Res 2015; 219: 65–82. 10.1016/bs.pbr.2015.03.003 26072234

[9] Lorente De Nó R . Studies on the structure of the cerebral cortex. II. Continuation of the study of the ammonic system. J Psychol Neurol 1934; 46: 113–77.

[10] Amaral DG, Witter MP. The three-dimensional organization of the hippocampal formation: a review of anatomical data. Neuroscience 1989; 31: 571–91. 10.1016/0306-4522(89)90424-7 2687721

[11] Ishizuka N, Weber J, Amaral DG. Organization of intrahippocampal projections originating from CA3 pyramidal cells in the rat. J Comp Neurol 1990; 295: 580–623. 10.1002/cne.902950407 2358523

[12] Palomero-Gallagher N, Kedo O, Mohlberg H et al. Multimodal mapping and analysis of the cyto- and receptorarchitecture of the human hippocampus. Brain Struct Funct 2020; 225: 881–907. 10.1007/s00429-019-02022-4 31955294 PMC7166210

[13] Blackstad TW . Commissural connections of the hippocampal region in the rat, with special reference to their mode of termination. J Comp Neurol 1956; 105: 417–537. 10.1002/cne.901050305 13385382

[14] Lein ES, Hawrylycz MJ, Ao N et al. Genome-wide atlas of gene expression in the adult mouse brain. Nature 2007; 445: 168–76. 10.1038/nature05453 17151600

[15] Fanselow MS, Dong HW. Are the dorsal and ventral hippocampus functionally distinct structures? Neuron 2010; 65: 7–19. 10.1016/j.neuron.2009.11.031 20152109 PMC2822727

[16] Qiu S, Hu Y, Huang Y et al. Whole-brain spatial organization of hippocampal single-neuron projectomes. Science 2024; 383: eadj9198. 10.1126/science.adj9198 38300992

[17] Bienkowski MS, Bowman I, Song MY et al. Integration of gene expression and brain-wide connectivity reveals the multiscale organization of mouse hippocampal networks. Nat Neurosci 2018; 21: 1628–43. 10.1038/s41593-018-0241-y 30297807 PMC6398347

[18] Cembrowski MS, Spruston N. Heterogeneity within classical cell types is the rule: lessons from hippocampal pyramidal neurons. Nat Rev Neurosci 2019; 20: 193–204. 10.1038/s41583-019-0125-5 30778192

[19] Cembrowski MS, Wang L, Sugino K et al. Hipposeq: a comprehensive RNA-seq database of gene expression in hippocampal principal neurons. eLife 2016; 5: e14997. 10.7554/eLife.14997 27113915 PMC4846374

[20] Yao Z, van Velthoven CTJ, Nguyen TN et al. A taxonomy of transcriptomic cell types across the isocortex and hippocampal formation. Cell 2021; 184: 3222–41. 10.1016/j.cell.2021.04.021 34004146 PMC8195859

[21] Batiuk MY, Martirosyan A, Wahis J et al. Identification of region-specific astrocyte subtypes at single cell resolution. Nat Commun 2020; 11: 1220. 10.1038/s41467-019-14198-8 32139688 PMC7058027

[22] Ayhan F, Kulkarni A, Berto S et al. Resolving cellular and molecular diversity along the hippocampal anterior-to-posterior axis in humans. Neuron 2021; 109: 2091–105. 10.1016/j.neuron.2021.05.003 34051145 PMC8273123

[23] Franjic D, Skarica M, Ma S et al. Transcriptomic taxonomy and neurogenic trajectories of adult human, macaque, and pig hippocampal and entorhinal cells. Neuron 2022; 110: 452–69. 10.1016/j.neuron.2021.10.036 34798047 PMC8813897

[24] Chen A, Liao S, Cheng M et al. Spatiotemporal transcriptomic atlas of mouse organogenesis using DNA nanoball-patterned arrays. Cell 2022; 185: 1777–92. 10.1016/j.cell.2022.04.003 35512705

[25] Chen A, Sun Y, Lei Y et al. Single-cell spatial transcriptome reveals cell-type organization in the macaque cortex. Cell 2023; 186: 3726–43. 10.1016/j.cell.2023.06.009 37442136

[26] Fang R, Xia C, Close JL et al. Conservation and divergence of cortical cell organization in human and mouse revealed by MERFISH. Science 2022; 377: 56–62. 10.1126/science.abm1741 35771910 PMC9262715

[27] Moffitt JR, Bambah-Mukku D, Eichhorn SW et al. Molecular, spatial, and functional single-cell profiling of the hypothalamic preoptic region. Science 2018; 362: eaau5324. 10.1126/science.aau5324 30385464 PMC6482113

[28] Tosches MA, Yamawaki TM, Naumann RK et al. Evolution of pallium, hippocampus, and cortical cell types revealed by single-cell transcriptomics in reptiles. Science 2018; 360: 881–8. 10.1126/science.aar4237 29724907

[29] Dugas-Ford J, Rowell JJ, Ragsdale CW. Cell-type homologies and the origins of the neocortex. Proc Natl Acad Sci USA 2012; 109: 16974–9. 10.1073/pnas.1204773109 23027930 PMC3479531

[30] Bakken TE, Jorstad NL, Hu Q et al. Comparative cellular analysis of motor cortex in human, marmoset and mouse. Nature 2021; 598: 111–9. 10.1038/s41586-021-03465-8 34616062 PMC8494640

[31] Wang Q, Ding SL, Li Y et al. The Allen Mouse Brain Common Coordinate Framework: a 3D reference atlas. Cell 2020; 181: 936–53. 10.1016/j.cell.2020.04.007 32386544 PMC8152789

[32] Paxinos G, Watson CRR, Petrides M et al. The Marmoset Brain in Stereotaxic Coordinates. New York: Elsevier, 2012.

[33] Paxinos G, Franklin KBJ. Paxinos and Franklin’s the Mouse Brain in Stereotaxic Coordinates. New York: Elsevier Academic Press, 2019.

[34] Paxinos G, Huang XF, Toga AW. The Rhesus Monkey Brain in Stereotaxic Coordinates. New York: Elsevier Academic Press, 2000.

[35] Liu C, Ye FQ, Newman JD et al. A resource for the detailed 3D mapping of white matter pathways in the marmoset brain. Nat Neurosci 2020; 23: 271–80. 10.1038/s41593-019-0575-0 31932765 PMC7007400

[36] Letzkus JJ, Wolff SBE, Lüthi A. Disinhibition, a circuit mechanism for associative learning and memory. Neuron 2015; 88: 264–76. 10.1016/j.neuron.2015.09.024 26494276

[37] Yao Z, van Velthoven CTJ, Kunst M et al. A high-resolution transcriptomic and spatial atlas of cell types in the whole mouse brain. Nature 2023; 624: 317–32. 10.1038/s41586-023-06812-z 38092916 PMC10719114

[38] Su Y, Zhou Y, Bennett ML et al. A single-cell transcriptome atlas of glial diversity in the human hippocampus across the postnatal lifespan. Cell Stem Cell 2022; 29: 1594–610. 10.1016/j.stem.2022.09.010 36332572 PMC9844262

[39] Zhou Y, Su Y, Li S et al. Molecular landscapes of human hippocampal immature neurons across lifespan. Nature 2022; 607: 527–33. 10.1038/s41586-022-04912-w 35794479 PMC9316413

[40] Gómez de San José N, Goossens J, Al Shweiki MR et al. Glutamate receptor 4 as a fluid biomarker for the diagnosis of psychiatric disorders. J Psychiatr Res 2022; 156: 390–7. 10.1016/j.jpsychires.2022.10.010 36323141

[41] Zhou H, Cheng Z, Bass N et al. Genome-wide association study identifies glutamate ionotropic receptor GRIA4 as a risk gene for comorbid nicotine dependence and major depression. Transl Psychiatry 2018; 8: 208. 10.1038/s41398-018-0258-8 30287806 PMC6172277

[42] Swanson LW, Cowan WM. An autoradiographic study of the organization of the efferent connections of the hippocampal formation in the rat. J Comp Neurol 1977; 172: 49–84. 10.1002/cne.901720104 65364

[43] Pitkänen A, Pikkarainen M, Nurminen N et al. Reciprocal connections between the amygdala and the hippocampal formation, perirhinal cortex, and postrhinal cortex in rat. A review. Ann NY Acad Sci 2000; 911: 369–91. 10.1111/j.1749-6632.2000.tb06738.x 10911886

[44] Cembrowski MS, Phillips MG, DiLisio SF et al. Dissociable structural and functional hippocampal outputs via distinct subiculum cell classes. Cell 2018; 173: 1280–92. 10.1016/j.cell.2018.03.031 29681453

[45] Li Y, Xu J, Liu Y et al. A distinct entorhinal cortex to hippocampal CA1 direct circuit for olfactory associative learning. Nat Neurosci 2017; 20: 559–70. 10.1038/nn.4517 28263300

[46] Lee H, Wang C, Deshmukh SS et al. Neural population evidence of functional heterogeneity along the CA3 transverse axis: pattern completion versus pattern separation. Neuron 2015; 87: 1093–105. 10.1016/j.neuron.2015.07.012 26298276 PMC4548827

[47] Jimenez JC, Su K, Goldberg AR et al. Anxiety cells in a hippocampal-hypothalamic circuit. Neuron 2018; 97: 670–83. 10.1016/j.neuron.2018.01.016 29397273 PMC5877404

[48] Lee SH, Marchionni I, Bezaire M et al. Parvalbumin-positive basket cells differentiate among hippocampal pyramidal cells. Neuron 2014; 82: 1129–44. 10.1016/j.neuron.2014.03.034 24836505 PMC4076442

[49] Mizuseki K, Diba K, Pastalkova E et al. Hippocampal CA1 pyramidal cells form functionally distinct sublayers. Nat Neurosci 2011; 14: 1174–81. 10.1038/nn.2894 21822270 PMC3164922

[50] Soltesz I, Losonczy A. CA1 pyramidal cell diversity enabling parallel information processing in the hippocampus. Nat Neurosci 2018; 21: 484–93. 10.1038/s41593-018-0118-0 29593317 PMC5909691

[51] Zhang W, Li SS, Han Y et al. Sex differences in electrophysiological properties of mouse medial preoptic area neurons revealed by *in vitro* whole-cell recordings. Neurosci Bull 2021; 37: 166–82. 10.1007/s12264-020-00565-9 32888180 PMC7870743

[52] Simonnet J, Eugène E, Cohen I et al. Cellular neuroanatomy of rat presubiculum. Eur J Neurosci 2013; 37: 583–97. 10.1111/ejn.12065 23176296

[53] Abbasi S, Kumar SS. Electrophysiological and morphological characterization of cells in superficial layers of rat presubiculum. J Comp Neurol 2013; 521: 3116–32. 10.1002/cne.23365 23681479

[54] Zhu JJ, Esteban JA, Hayashi Y et al. Postnatal synaptic potentiation: delivery of GluR4-containing AMPA receptors by spontaneous activity. Nat Neurosci 2000; 3: 1098–106. 10.1038/80614 11036266

[55] Loomba S, Straehle J, Gangadharan V et al. Connectomic comparison of mouse and human cortex. Science 2022; 377: eabo0924. 10.1126/science.abo0924 35737810

[56] Francavilla R, Villette V, Luo X et al. Connectivity and network state-dependent recruitment of long-range VIP-GABAergic neurons in the mouse hippocampus. Nat Commun 2018; 9: 5043. 10.1038/s41467-018-07162-5 30487571 PMC6261953

