# Supplementary material for: Evolutionary convergence and divergence of hippocampal cytoarchitecture between rodents and primates revealed by single-cell spatial transcriptomics
Source: Natl Sci Rev. 2026 Jan 2;13(5):nwaf595. doi: 10.1093/nsr/nwaf595 (PMC12949517; doi:10.1093/nsr/nwaf595)
Supplement: nwaf595_Supplemental_Files [file nwaf595_supplemental_files.zip › supplementary methods.docx]

Methods

Animals

Animals were housed under a 12 h light / dark cycle and provided with food and water *ad libitum* in the animal facility of Institute of Neuroscience (CAS Center for Excellence in Brain Science and Intelligence Technology). All animal procedures (ION-2019011, CEBSIT-2021038, CEBSIT-2021039, NA-047-2020) were performed in accordance with institutional guidelines and were approved by the Institutional Animal Care and Use Committee (IACUC) of the Institute of Neuroscience, CEBSIT, CAS.

Three male cynomolgus monkeys (*M. fascicularis*; #1, 6-year-old, 4.2 kg; #2, 4-year-old, 3.7 kg; #3, 7-year-old, 10.6 kg), these animals were also used for study on cortical transcriptome^25^. Two male common marmosets (Callithrix jacchus; #1, 3-year-old, 458 g; #2: 7-year-old, 321 g), and two male mice (C57BL/6J, 11-week-old, male) were used for Stereo-seq and snRNA-seq experiments.

The marmosets that were used for slice recording were diagnosed with digestive problems such as diarrhea in the animal facility and had to be euthanized based on the animal welfare regulation, but their brain samples could be used for slice recording. Three marmosets were used for slice recording of pyramidal cells in CA3 and CA4 (male, 321g, 3-year-old; male 210 g, 3-year-old; female 230g, 6-year-old), and five marmosets were used for slice recording of pyramidal cells in CA1 (in age, sex, weight: 1 year, female, 238 g; 3 years, male, 277 g; male, 5 years, 273 g; male, 5 years, 221 g; female, 7 years, 320 g). Furthermore, ten male mice (C57BL/6J, 11-12 weeks old) were used for slice recording.

Human tissues

Hippocampal tissue blocks were provided by the Netherlands Brain Bank (Amsterdam, The Netherlands, www.brainbank.nl). All donors provided informed consent for brain autopsy and the use of their tissue and clinical data for research purposes in compliance with Dutch national ethics guidelines. Additional ethical screening and approval for using post-mortem human brain tissue for molecular profiling was provided by the regional ethical committee in (EPN, Stockholm, Sweden, 2013/474-31/2).

We obtained three hippocampal sections from three human subjects (1#, male, 79 years old, PMI 6 h; 2#, female, 81 years old, PMI 7.5 h; female, 79 years old, PMI 6 h). One hippocampal section was not complete because its CA1 and CA3 were partially cut away.

Tissue collections from macaques, marmosets and mice

The animals were deeply anesthetized with 3% isoflurane and the brain was quickly perfused with 4℃ artificial cerebrospinal fluid (ACSF) bubbled with oxygen (influx with a mixture of 95% O_2_ and 5% CO_2_). The whole left hemisphere was obtained along the brain raphe using the mouse brain slicers (RWD, #68708). The isolated brain blocks were quickly wiped dry with sterile gauze and rolled three times in a container covered with OCT (4583#, Sakura) to adequately displace the water attached to the brain mass to prevent the ice crystals during sectioning. Subsequently the brain blocks were transferred to a self-made metal mold with OCT at the bottom, and OCT was added to fully cover the brain blocks. The brain blocks were quick-frozen with dry ice powder, marked, and then stored in -80°C refrigerator. To minimize RNA degradation, all solutions were prepared with sterilized water containing diethyl pyrocarbonate (DEPC) (B501005-0005, Sangon Biotech), and all instruments were washed with sterilized water containing DEPC and RNase Zap (AM9780, Invitrogen). The whole tissue collection process was completed within 10 min.

The Stereo-seq data acquisition from macaques, marmosets and mice

***Tissue preparations*** Before cryosection, temperature of the cryochamber was set to -20℃ and the specimen head was set to -12℃ (Thermo Fisher Cryostar NX50). The tools involved (chip, forceps, brush and blade) were placed in the cryochamber in advance for pre-cooling. The tissue blocks were equilibrated in the cryochamber at least one hour in advance. At each desired coronal coordinate, cryosection was performed to obtain one 10-μm section for Stereo-seq at 100-μm interval. Between successive days of sectioning, the tissue blocks were stored at -80℃. The thin Stereo-seq sections were firstly flattened on blade holder base (-20℃) by soft brush and plastic tweezers. Then the section was carefully placed on a pre-cooled Stereo-seq chip (-20℃) and moved from one end to the other with a finger gently pressed under the chip. The temperature of the finger kept the section close to the chip, reducing bubbles and wrinkles. In order to ensure the quality of samples, in addition to the right brain tissue after perfusion for total RNA extraction and quality detection, tissues trimmed between two chips during sectionalization will also be collected for total RNA extraction and quality detection, and RNA RIN value greater than or equal to 9 will be regarded as qualified samples.

***Stereo-seq chips*** The tissue section on the Stereo-seq chip (1 cm x 1 cm) was then baking at 37℃ for 5 min and subsequently fixed in methanol (Sigma, 34860, precooled for 30 mins at -20℃; 2 ml methanol was added in multi-orifice for each section) and incubated at -20℃ for 30 min. Methanol was then dried out in a hood. Tissue section on the chip was then stained with ssDNA reagent (Invitrogen, Q10212) for 5 min and subsequently washed with 0.1x SSC buffer (Ambion, AM9770; containing 0.05 U/μl RNase inhibitor). Section images were captured using Zeiss Axio Scan Z1 microscope (at EGFP and DAPI wavelength, 10-ms exposure). Tissue sections were then permeated by incubating in 0.1% pepsin (Sigma, P7000) at 37℃ for 12 min (pepsin was prewarmed at 37℃ for 3 min) in 0.01M HCl buffer (pH 2) and then washed with 0.1×SSC buffer (containing 0.05 U/μl RNase inhibitor) to remove pepsin. In this step, RNAs were released from the permeated tissue and captured by Stereo-seq chip. RNAs were then reverse transcribed for 2 hours at 42℃. After reverse transcription, tissue sections were washed with 0.1× SSC buffer and digested with tissue removal buffer (10 mM Tris-HCl, 25 mM EDTA, 100 mM NaCl, 0.5% SDS) at 55℃ for 30 min, and then the chips were washed twice with 0.1× SSC buffer. The cDNA-containing chips were then subjected to Exonuclease (NEB, M0293L) treatment for 1 hour at 37℃ and were washed once with 0.1× SSC buffer. The cDNAs were amplified with Hot Start DNA Polymerase (QIAGEN). The PCR reaction protocol was first incubation at 95℃for 5 min, 15 cycles at 98℃ for 20 s, 58℃ for 20 s, 72℃ for 3 min and a final incubation at 72℃for 5 min. The PCR products were then purified using 0.6× VAHTSTM DNA Clean Beads and were quantified by Qubit dsDNA HS assay kit (Invitrogen, Q32854).

***Library preparation and sequencing*** The library preparation were processed as described previously^25^. In brief, cDNA from each sample were dealt with Tn5 transposases (Vazyme) at 55℃ for 10 mins, then the reaction was stopped by adding 0.02% SDS. PCR reaction mix (Library HIFI Master Mix, Library PCR primer mix) was added to each fragmented cDNA sample. Samples were then transferred to a thermal cycler for amplification using the following protocol: 1 cycle at 95℃ for 5 min, 13 cycles of tri-temperature reaction (98℃ 20 s, 58℃ 20 s and 72℃ 30 s), and 1 cycle at 72℃ for 5 min. After amplification, the PCR products were purified with 0.6× and 0.2× VAHTSTM DNA Clean Beads (VAZYME, N411-03) and were used for DNB (DNA Nano Ball) generation. Finally, the DNBs were sequenced on the DNBSEQTM T10 sequencing platform (MGI, Shenzhen, China) with 50 bp read1 and 100 bp read2.

The Stereo-seq data acquisition from humans

The stereo-seq data acquisition was largely same as those for macaques, marmosets and mice, but there were some differences as follows: (1) Following staining with the ssDNA reagent, section images were captured using a VSlide scanning microscope (at EGFP wavelength). (2) Reverse transcription was for 3-4 hours at 42℃. (3) After reverse transcription, tissue sections were digested with tissue removal buffer (MGI) at 55℃ for 10-20 min, and then the chips were subjected to treatment with cDNA release mix (MGI) overnight at 55℃. (4) For library preparation, after amplification, the PCR products were purified with 0.55× and 0.15× VAHTSTM DNA Clean Beads. (5) The DNBs were sequenced on the DNBSEQTM T10 sequencing platform (MGI, Riga, Latvia).

Stereo-seq data analysis

***Data preprocessing*** FastQ files were generated using a MGI DNBSEQ-T20 sequencer. Coordinate Identity (CID) sequences and Molecular Identifier (MID) sequences were contained in the read 1 (CID: 1-25 bp, MID: 26-35 bp) while the read 2 consist of the cDNA sequences. CID sequences on the first reads were first mapped to the designed coordinates of the in situ captured chip achieved from the first round of sequencing, allowing 1 base mismatch to correct for sequencing and PCR errors. Reads with MID containing either N bases or more than 2 bases with quality score lower than 10 were filtered out. CID and MID associated with each read were appended to each read header. Retained reads were then aligned to the reference genome (Macaca_fascicularis_5.0 for macaca, Callithrix_jacchus_cj1700_1.1 for marmoset) using STAR (v2.7.4a) and mapped reads with MAPQ > 10 were counted and annotated to their corresponding genes. UMI with the same CID and the same gene locus were collapsed, allowing 1 mismatch to correct for sequencing and PCR errors. Finally, this information was used to generate a CID-containing expression profile matrix. The whole procedure was integrated into a publicly available pipeline SAW available at <https://github.com/BGIResearch/SAW>.

***Expression profile matrices*** were first divided into non-overlapping bins of area 50×50 DNBs (bin50, 25 µm resolution). Reads of the same gene were summed up within each bin. In order to reduce variance and utilize spatial information, raw counts from each bin were smoothed. This step is equivalent to applying a gaussian filter to the 2D expression matrix on each gene. After smoothing, counts in each bin50 were no longer integers, which makes some normalization methods such as SCTransform unsuitable. In this way, the conventional method, log cpm, was used to normalize smoothed counts. Counts from each bin after smoothing were normalized to a total sum of 1000,000. A pseudo count of 1 was added to each gene and followed by logarithmization. The logarithmized counts were used for subsequent analysis.

***Transcriptome-based subregion classifications using Stereo-seq data*** Clustering was first carried out on each coronal section. Then clusters from each section were aligned together as domains. Python package Scanpy was used for the first stage. We used the Scanpy function pp.neighbors to find neighbors for each bin50. Since counts have already been smoothed in our pipeline, the expression levels already contained spatial information. By adjusting the sigma parameter for smoothing, weights were automatically handled between spatial and transcriptomic information. The k-neighbor graph was then used as input for leiden clustering. Marker genes were called using the Wilcoxon rank sum test provided by the rank_genes_groups function.

To align clusters from different sections, prior knowledge, mainly anatomical features, was first used to define domains on one section. Clusters on a second section were matched to the first section using Pearson correlation between expression vectors as weights. This procedure could be viewed as maximum bipartite matching and is handled by python package networkx. Then unassigned clusters were merged according to expression similarity with neighboring domains. Clusters of poor quality or some mis-assigned clusters were then corrected manually. These subregion classifications were also validated using other tools including BANKSY and GraphST.

***Drawing of conventional atlas-based hippocampal subregions*** Conventional atlas-based hippocampal subregions on each section were drew on the total RNA images based on the Paxinos atlases ^32-34^ using the ITKsnap software^57^. First, the total RNA images of each section were converted into NIFTI format (<https://nifti.nimh.nih.gov/nifti-2)> using in-house developed python script and NiBabel python package and loaded into ITKsnap (<https://nipy.org/nibabel/nifti_images.html>). Second, the subregions CA1, CA2, CA3, CA4, DG as well as CA1 stratum layers were drew based on atlases. To identify the CA2 subregion, we converted the expression patterns of marker gene *PCP4* into NIFTI images to guide the drawing. Prosubiculum and subiculum were not distinguishable in total RNA images and drew together as SUB. Presubiculum and parasubiculum were not distinguishable in total RNA images and drew together as pSUB. Finally, all the manually drawn subregions were converted back into the corresponding matrix of spatial transcriptome data for further analysis.

The snRNA-seq data acquisition

***Tissue collection and processing for snRNA-seq*** The hippocampal tissues of two macaques, one marmoset and two mice were used for snRNA-seq. Hippocampal tissues were extracted from the tissue block using tissue punchers and forceps. The collected tissues were then rapidly frozen in liquid nitrogen and stored in pre-cooled tubes at -80°C before snRNA-seq sequencing.

***snRNA-seq library construction and sequencing*** The DNBelab C Series Single-Cell Library Prep Set was used as previously described^58^. In brief, single-nucleus/cell suspensions were used for droplet generation, emulsion breakage, bead collection, reverse transcription and cDNA amplification to generate barcoded libraries. Indexed libraries were constructed according to the manufacturer’s protocol. Concentrations were measured with Qubit ssDNA Assay Kit (Thermo Fisher Scientific, Q10212). Libraries were sequenced on a DNBSEQ-T1 or DNBSEQ-T7 sequencer at the China National GeneBank (Shenzhen, China) with the following sequencing strategy: 41-bp read length for read 1 and 100-bp read length for read 2.

The snRNA-seq data analysis

***snRNA-seq data preprocessing*** The raw sequencing reads from DNBSEQ-T1 or DNBSEQ-T7 were initially processed using PISA (v0.2; https://github.com/shiquan/PISA) for filtration and demultiplexing. Following this, alignment to the Macaca_fascicularis_5.0 and Callithrix_jacchus_cj1700_1.1 genome was performed using STAR (v2.7.4a), and the resulting alignments were sorted using sambamba (v0.7.0). For tissues sequenced using snRNA-seq, a customized 'pre-mRNA' reference was created to appropriately align the count reads to both introns and exons. This was necessary due to the abundance of unspliced pre-mRNA within the cell nucleus. Consequently, gene transcript counts within snRNA-seq were obtained by combining counts from both exonic and intronic reads. Finally, PISA was employed to generate a UMI-based count matrix, linking cells/nuclei to gene expression levels.

***Cell clustering and cell type identification*** We used Seurat (v4.1 R package) and custom scripts to cluster and identify cell types from snRNA-seq data in three species. We obtained a total of 168,104 single nuclei from the hippocampus of three species, 81,638 from macaques, 33,667 from marmoset and 52,799 from mice. Single nuclei with number of genes (nFeature) less than 1000, the ratio of UMIs (nCounts) and nFeature less than 1.2, and the percentage of mitochondrial genes (percent.mt) more than 5% for each species was discarded. Then, we used SCT transformation to normalize gene expression levels with top 3000 highly variable genes in each dataset. Based on SCT normalized gene expression matrix, cell clustering analysis was performed by "RunPCA", "FindNeighbors", and "FindClusters" functions with parameters PC = 1:26, dimension= 1:15, resolution = 0.5 for macaque; PC = 1:41, dimension= 1:25, resolution = 0.8 for marmoset; PC = 1:30, dimension= 1:20, resolution = 0.5 for mouse. After unsupervised clustering, outlier cells per cluster and cells with mean expression of mitochondrial gene > 2 were removed.

The “FindAllMarkers” function was used to identify differentially expressed genes in each cluster. Then, based on the expression of classical marker genes, we annotated with specific cell types. Based on the expression of key marker genes, cells from the 3 species were annotated into 3 major cell classes: glutamatergic neurons (SLC17A7/6+), GABAergic neurons (GAD1/2+), non-neuronal cells (astrocytes: AQP4; S100B, and GFAP; oligodendrocytes: MOBP and PLP1; OPC: PDGFRA and VCAN; microglia: APBB1IP, PTPRC, Siglech, and Tmem119; endothelial: FLT1 and SPARC; VLMC: CEMIP). Then we extracted glutamatergic neurons and GABAergic neurons separately to perform PCA, followed by sub-clustering of the cells into subtypes using the “FindClusters” and “FindNeighbors” functions in Seurat, and annotated different subtypes based on canominal marker genes and their spatial distribution patterns.

***Integrative clustering across species*** To compare the similarity of cell types across macaques, marmosets and mice, we projected all cells from the 3 species in the same low dimension space and subsequently visualized them using UMAP in Seurat. Briefly, we downloaded the homologous gene list (version 9.1) for three species from ENSEMBL biomart database (https://www.ensembl.org/info/data/biomart/index.html), and expression data of homologous genes across three species were obtained for the subsequent analysis. For each species, we identified the top 3,000 highly variable genes using the SCT transformation (SCTransform function) and chose the features for integration using SelectIntegrationFeatures function. The anchors across species were subsequently identified using the function “FindIntegrationAnchors”. Then we scaled the integrated data, performed principal components analysis (PCA) (PCs 1:100) which were used for downstream UMAP visualization. Lastly, we independently mapped the annotation to UMAP. To quantify the cell type diversity across species, a co-clustering matrix was generated that represented the proportion of clustering in which each pair of nuclei was assigned to the same cluster.

***Differentially expressed genes (DEGs)*** Integrated Seurat objects were used to identify conserved and divergent subclass level DEGs across species. We firstly down-sampled 200 cells per subtype for each subclass, followed by “FindAllMarkers” function to identify DEGs for each subclass using the SCT normalized counts with Wilcoxon rank sum test. Eulerr package was used to generate Venn diagrams and Seurat’s DoHeatmap function was used to visualize conserved and divergent DEGs. We used clusterprofile to perform KEGG functional enrichment. Marker genes of cluster with p-value < 0.05 and log2FC > 0.5 were retained for KEGG pathway enrichments separately. Pathways with p-value < 0.05 were retained for the top 10 KEGG pathways.

Integration of Stereo-seq and snRNA-seq data

***Cell-type registration of Stereo-seq data*** We first segmented individual hippocampal cells on each Stereo-seq map as previously reported^25^, and then used Seurat and Cell2location to integrate Stereo-seq data with snRNA-seq data. We performed nuclei acid staining to mark single nuclei on each Stereo-seq section and adapted an automatic deep-learning model to segment single cells. This model was trained by images of the cerebral cortex in each species after plasma membrane stained by ConA ^25^, which marked boundaries between cells. We manually labeled one ConA image and used it for validation purposes. After manual correction, the ConA model was updated after addition of the newly labeled ConA image and repeated for several iterations for optimal validations.

Seurat was used for the registration of three major cell classes including glutamatergic and GABAergic neurons, and non-neuronal cells in the Stereo-seq map, and Cell2location was then used for the transfer of each cell type in each major cell class based on a Bayesian model that estimates the absolute abundance of cell types at each location by decomposing the spatial expression count matrix into a set of predefined reference cell type features. For major cell class registration, we used the SCTransform function to normalize the spatial transcriptome data, and filtered out mitochondria and ribosome-related genes. Then the FindTransferAnchors function was used to find anchors and predict cell class labels for each single cell on the spatial map, and the cell class with the highest prediction score was designated as the final cell class.

For subsequent cell type registration, we extracted gene expression matrices and cell types for each cell class from snRNA-seq and Stereo-seq data. Then we filtered mitochondria and ribosome related genes, and used *filter_genes* function to filter low quality genes with parameters: cell_count_cutoff = 15, cell_percentage_cutoff2 = 0.05, nonz_mean_cutoff = 1.12. The signatures were estimated from snRNA-seq data to account for potential batch effect using setup_anndata function with default parameters. Then we used Cell2location function to create and train a cell2location spatial mapping model with parameters: N_cells_per_location = 1, detection_alpha = 200. Finally, we exported the estimated cell abundance, and take a cell type with the highest abundance score as the identity for each cell in the Stereo-seq maps. Based on the spatial distribution patterns of various cell types, we re-grouped the cell types with overlapping or close proximity on the Stereo-seq map and UMAPs together into cell subclasses.

***Correspondence of cell types between stereo-seq and snRNA-seq data*** The snRNA-seq and single-cell Stereo-seq data were normalized using the SingleCellExperiment objects in R package Seurat. Then the two SingleCellExperiment objects were merged using the top variable genes as the var_genes parameter in the trainModel function of the MetaNeighbor package. Then a cell type-by-cell type mean AUROC matrix through the MetaNeighborUS function was computed showing the correspondence between the two omics data.

***Correlation of cell type distribution*** Between sections, two adjacent sections were selected to calculate the cell proportion of a single cell type (cell count of a specific cell type/total cell count of all cell types), and then Pearson analysis was used to calculate the similarity of cell type distributions among different sections. Between biological replicates, we performed similar analysis as above using two sections at similar coordinates.

***Spatial distribution of GABAergic cell types*** We firstly calculated the composition of various spatial transcriptome-defined subregions in each cell type though dividing cell number of each subregion by the total number of cells for each cell type. *P* value was calculated by scCODA ^59^ to assess the preferential distribution of specific subregions for each cell type. Cell types with compositions in a specific subregion more than 0.4 were further marked with asterisks.

***Cell type enrichment score calculation*** In order to assess both cell abundancy and aggregation, we used the normalized second moment of the cell density as the cell type enrichment score for downstream analysis. First, cell density was calculated on bin50 spatial matrices. The calculated cell density was then smoothed to further utilize spatial information. In this step, cell capture rate differences were normalized. Then the second moment of cell density was calculated. The second moment places a penalty on dispersed distributions which may measure cell enrichment better than simply using the mean cell density across the whole matrix.

***Separation of snRNA-seq data in CA2/3/4 fields using spatial marker genes*** We separated the spatial transcriptome profile of “CA2/3-pyr” into “CA2-pyr” and “CA3-pyr” based on conventional atlas-based hippocampal subregions, and used “FindMarkers” to identify spatial marker genes for CA2 and CA3 with p-value < 0.05 and log2FC > 0.5 from all sections of Stereo-seq data. Then, we performed further clustering analysis from snRNA-seq data of glutamatergic “Glu CA2/3” subclass in marmosets and macaques, and used the gene module of top 10 spatial marker genes to categorize the subclusters into glutamatergic “Glu CA2” and “Glu CA3” cell types, respectively. Using similar approaches, we separated snRNA-seq data of “Glu CA3/4" subclass in macaques and marmosets using spatial marker genes.

Finally, we used a subsampling method for DEG analysis from same number of neurons in each subclass. The volcano plots were used to show the DEGs between CA4 and CA3, CA2 and CA3 in macaque and marmoset glutamatergic cells, based on the separated snRNA-seq data of “Glu CA2”, “Glu CA3”, and “Glu CA4” subclasses in marmosets and macaques. The DEGs with p-value < 0.05 and log2FC > 0.5 were labeled in red dots.

***Transcription factor (TF) analysis*** Python package pySCENIC was used for gene regulatory network inference ^60^. We used the mouse TF list as reference to identify co-expression modules by the GRNBoost2 algorithm. Then we obtained the regulons by detecting the genes directly targeted by the TF and removing other genes based on the enrichment of motifs around the transcription start site using cisTarget databases. The human TF list was downloaded from (https://github.com/aertslab/pySCENIC/blob/master/resources/hs_hgnc_tfs.txt), and the cisTarget database was downloaded from (<https://resources.aertslab.org/cistarget>). Using aucell, the regulon activity score was measured as the area under the recovery curve. The regulon specificity score (RSS) was used to detect the cell type-specific regulons ^61^. The TFs with RSS score exceeding 0.1 were identified specificity for illustrating the heatmap. The ZSCNACE(-) indicates that ZSCNACE is a transcription factor with suppressive activity.

***Spatial neighborhood analysis*** Neighborhood enrichment score between cell type A and cell type B was defined as the log ratio between the average percentage of cell type B within the neighborhood of cell type A and the average percentage of cell type B for all cells types. The specific approach of MetaNeighbor is to first calculate the correlation between all cell pairs based on the expression of a set of genes and construct a cell network; then, through cross-validation, the cell type labels of one dataset are hidden and used as the test set, and the other datasets are used as the training set; finally, the neighbor voting algorithm is used to predict the cell type labels of cells in the test set based on the similarity between the cells in the training set and the cells in the training set. We calculated the neighborhood enrichment score for each cell type on each Stereo-seq section with Z-test. For each cell, we only considered its nearest 32 cells as its neighbors. A pseudo-count of 1 is added when calculating cell proportions to avoid zeros during logarithm.

***Spatial cell-cell interaction analysis*** We only took into account interactions between glutamate & glutamate receptors and GABA & GABA receptors in this section. The interaction strength was measured by the product between the ligand and receptor expression levels in the single cell dataset. To further assess the significance of such interaction, we considered neighboring cell pairs in the spatial datasets. Neighboring cells were defined as the same as the section above. We assumed all GABAergic cells expressed *GAD1* and all glutamatergic cells express *GLS*. The interaction score was measured by the weighted average of gene expression of these receptors. The weights are the cell distributions used in the spatial neighborhood analysis. To further assess the significance of such interaction, we calculated spearman correlation as the neighboring score between cell and gene expression distributions.

***Gene gradients along superficial-deep and proximal-distal axes***. We first calculated superficial-deep and proximal-distal coordinates in CA1 sections of all three species, and then carried out spearman correlation tests between gene expression and these coordinates in the CA1-pyr subregion (*str. pyramidale*). The distribution offset was then determined by the distance between the averaged Z-scored coordinates of all cells and the CM in each subregion. Genes with distribution offsets>0.2 and higher expression levels inside CA1-pyr than outside CA1-pyr and were marked with asterisks and sorted according to the averaged spearman correlation across all sections in each species.

FISH assay

To design a hybridization probe, the target sequence region with high hybridization efficiency and strong specificity is screened from the RNA sequence of the cell or tissue to be detected. A reaction chamber was prepared on the sample. The PFA-fixed samples were soaked in 70%, 80%, and 100% gradient alcohol (5 min each time), washed twice with D-PBST, treated with 0.1M HCl for 5 min, washed twice with PBST. The reaction was incubated at 37 °C overnight with hybridization buffer, washed three times with PBST, and reacted at 16 °C for 3h with the ligase system added. The ligase reaction system was washed with PBST several times before adding the rolling circle amplification reaction system. The rolling circle amplification reaction system was incubated at 30 ℃ overnight and washed with PBST. Furthermore, the fluorescent probe hybridization system was applied for in situ hybridization. Finally, the slides were dehydrated with gradient alcohols and protected by coverslips. The area of interest was imaged by a Leica thunder image system and the RNA spatial information was analyzed in ImageJ. The FISH assay for each gene was repeated for 2 - 3 times.

Slice recording and analysis

Mice and marmosets were anaesthetized by isoflurane (5%) and then deeply anaesthetized by high-dose isoflurane (~150 µL in custom nose masks modified from 15- and 50-mL falcon tubes, respectively). Animals were transcardially perfused with ice-cold NMDG-based slicing artificial cerebral spinal fluid (ACSF, 93 mM NMDG, 2.5 mM KCl, 1.2 mM NaH_2_PO_4_, 30 mM NaHCO_3_, 20 mM HEPES, 5 mM Sodium ascorbate, 2 mM thiourea, 3 mM Sodium pyruvate, 25 mM D-glucose,12 mM N-Acetyl-L-cysteine, 10 mM MgCl_2_, 0.5 mM CaCl_2_, oxygenated with 95% O_2_/5% CO_2_). The brain was immediately removed after the perfusion, and transferred to ice-cold NMDG-based slicing ACSF. Coronal brain slices (300 µm thick) containing hippocampus were prepared using a vibratome (VT-1200S, Leica) in an ice-cold NMDG-based ACSF. Slices were maintained for 12 min at 32°C in NMDG-based ACSF and were subsequently transferred into HEPES-based solution containing (in mM: 92 NaCl, 2.5 KCl,1.2 NaH_2_PO_4_, 30 NaHCO_3_, 20 HEPES, 5 Sodium ascorbate, 2 thiourea, 3 Sodium pyruvate, 25 D-glucose, 2 MgCl_2_, 2 CaCl_2_, bubbled with 95% O_2_/5% CO_2_) at room temperature and incubated more than 1 h before recording, and then were kept at room temperature (20-22°C) until start of recordings_._ For the recording, slices were transferred to a recording chamber and infused with ~30 °C recording ACSF (in mM: 119 NaCl, 5 KCl, 1.25 NaH_2_PO_4_, 26 NaHCO_3_, 10 D-glucose, 1 MgCl_2_, 2 CaCl_2_, bubbled with 95% O_2_/5% CO_2_). All chemicals were purchased from Sigma-Aldrich. Patch pipettes (4 – 7 MΩ) pulled from borosilicate glass (Sutter instrument, BF150-86-10) were filled with a K-gluconate based internal solution (in mM: 126 K-gluconate, 2 KCl, 2 MgCl_2_,10 HEPES, 0.2 EGTA, 4 MgATP_2_, 0.4 Na_3_GTP, 10 Na-phosphate creatine, 290 mOsm, adjusted to pH 7.2~7.3 with KOH). Whole-cell recording was performed with a Multiclamp 700B amplifier and a Digidata 1440A (Molecular Device). The data was analyzed by Clampfit. The ∆G_sag_ is quantified as the change in membrane conductance by 100×(G_hyp_- G_sag_)/ G_sag_, is known to indicate the I_h_ ^51^.

**Supplementary references**

57. Yushkevich, P.A.*, et al.* User-guided 3D active contour segmentation of anatomical structures: significantly improved efficiency and reliability. *Neuroimage* **31**, 1116-1128 (2006).

58. Han, L.*, et al.* Cell transcriptomic atlas of the non-human primate Macaca fascicularis. *Nature* **604**, 723-731 (2022).

59. Buttner, M., Ostner, J., Muller, C.L., Theis, F.J. & Schubert, B. scCODA is a Bayesian model for compositional single-cell data analysis. *Nat Commun* **12**, 6876 (2021).

60. Aibar, S.*, et al.* SCENIC: single-cell regulatory network inference and clustering. *Nat Methods* **14**, 1083-1086 (2017).

61. Suo, S.*, et al.* Revealing the Critical Regulators of Cell Identity in the Mouse Cell Atlas. *Cell Rep* **25**, 1436-1445 e1433 (2018).

62. Ma, S.*, et al.* Molecular and cellular evolution of the primate dorsolateral prefrontal cortex. *Science* **377**, eabo7257 (2022).
