# Supplementary material for: Evolutionary convergence and divergence of hippocampal cytoarchitecture between rodents and primates revealed by single-cell spatial transcriptomics
Source: Natl Sci Rev. 2026 Jan 2;13(5):nwaf595. doi: 10.1093/nsr/nwaf595 (PMC12949517; doi:10.1093/nsr/nwaf595)

Figure S1. The characteristics of spatial transcriptome-defined subregions in the hippocampus

(**A**) Jaccard similarity of marker genes for spatial transcriptome-defined subdomains across different coronal sections in macaque, marmoset and mouse, revealing high similarity for the same subregion across different sections in each species.

(**B**) Spatial clustering of the Stereo-seq data from biological replicates, showing the reliability across animal replicates as well as the consistency of spatial transcriptome-defined hippocampal subregions across species. The color codes for spatial transcriptome-defined subregions were the same as those in C. Scale bars, 1 mm.

(**C**) Sanky plot showing the correspondence between spatial transcriptome-defined subregions and conventional histology-defined subregions in the hippocampus of all three species.

(**D**) Dot plot showing marker genes of laminar structures in the macaque (upper) and marmoset (lower) subiculum complex.

(**E**) Heatmap showing the similarity of spatial transcriptome-defined subregions across species. The similarity was quantified by Pearson correlation coefficients of top 1500 variable genes in each cluster. Asterisks indicate the highest correlation between subregions of two species.

(**F**) Venn plot showing shared marker genes among macaque, marmoset and mouse for each spatial transcriptome-defined subregion.

(**G**) Human hippocampal subregions defined by unsupervised clustering analysis of Stereo-seq data. The subregions were color-coded with legends shown at right. Scale bars, 1 mm.

(**H**) Spatial expression patterns of CA1 marker genes *FIBCD1* (magenta) and *NTS* (green) validated by FISH in hippocampal sections from marmoset replicates, respectively. Scale bar, 0.5 mm.

(**I**) Correlation of cell-type distributions between sections at similar coordinates from different animal replicates in the same species. The coordinate and animal number were labeled in the title of X and Y axes.


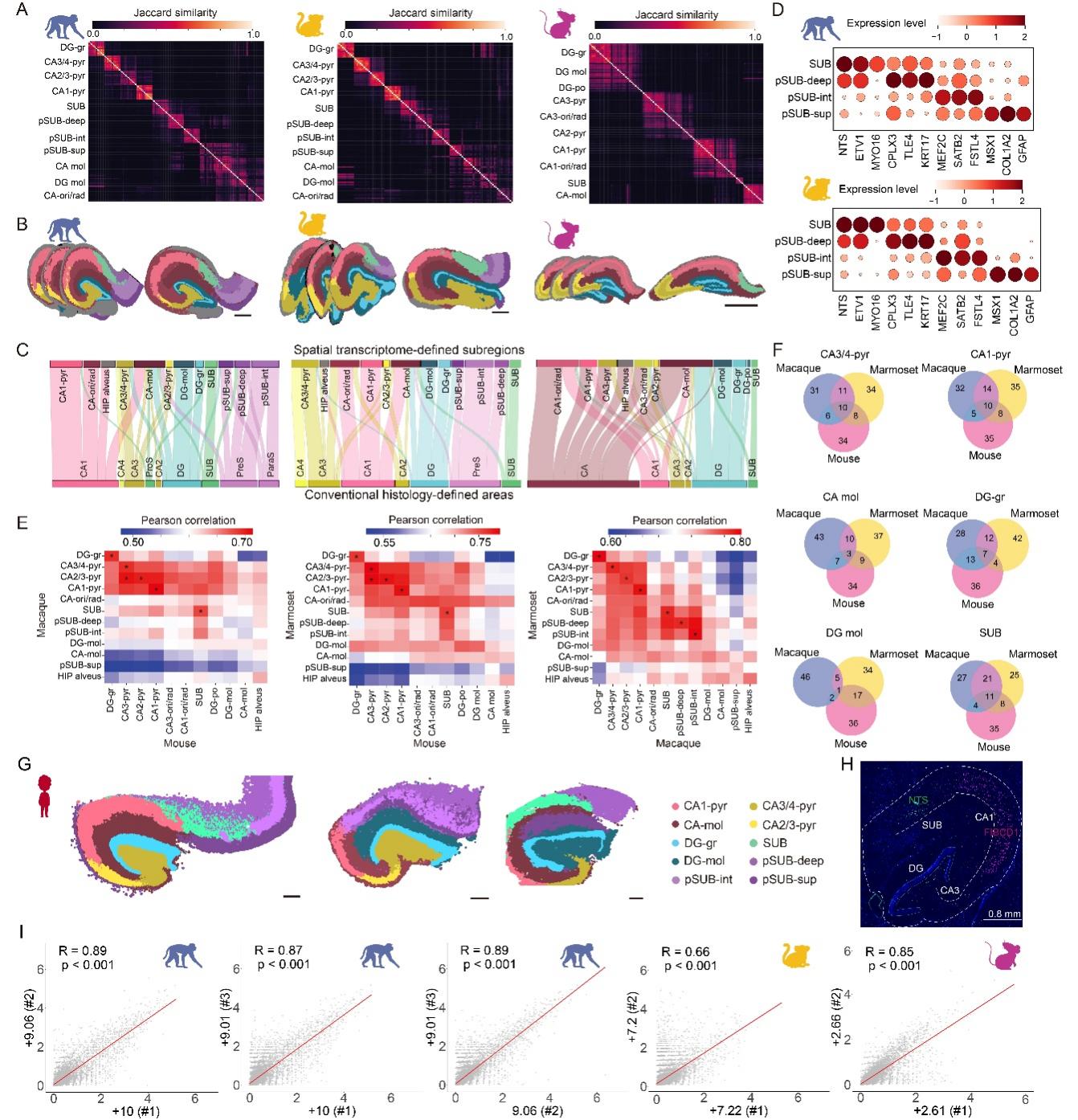


Figure S2. The reliability and spatial registration of cell types defined by snRNA-seq clusters.

(**A**) Proportion of species composition across glutamatergic neuron subclasses. Note Glu CA2 and CA3 subclasses were only found in mice.

(**B**) The UMAP plot of subclasses using 40%, 60%, and 80% down-sampled snRNA-seq data of glutamatergic neurons in the macaque hippocampus. The number of subclasses in all down-sampling analysis were the same (n = 19), indicating the sufficiency of cell numbers in macaques for cell typing.

(**C**) The UMAP showing unsupervised clustering results in macaques, marmosets and mice.

(**D**) Violin plots showing the number of UMI counts, number of genes and percent of mitochondrial genes in each snRNA-seq-defined cell cluster of three species.

(**E**) Correlation of cell-type distributions between two adjacent sections from the same animals (left) and between sections at similar coordinates from different animals (right).

(**F**) Relationship between correlation coefficients of cell-type distributions and distances between sections (in mm).

(**G**) Heatmaps showing the similarity of gene expression profiles between cell types in Stereo-seq data after single-cell registration (Stereo-seq cell types) and snRNA-seq data (snRNA-seq cell types).


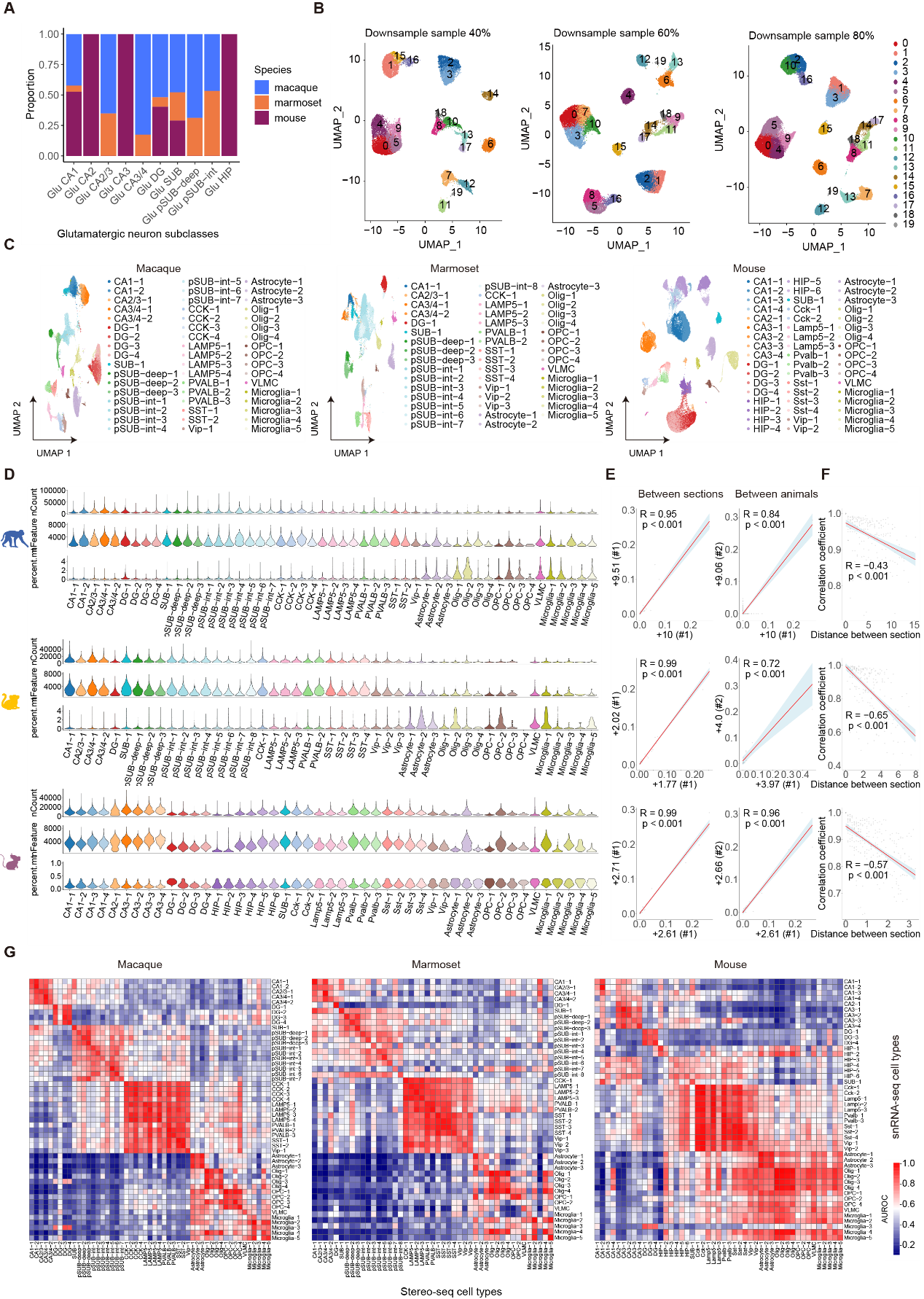


Figure S3. The spatial distribution of cell subclasses and FISH assay in biological replicates.

(**A**) FISH validation for the spatial expression of CA1, CA3/4 and DG marker genes *MAN1A1*, *TRPS1*, and *RFX3* in animal replicates, respectively. Scale bars, 1 mm.

(**B**) Spatial distribution of various cell types in Stereo-seq maps along the A-P axis from biological replicates of the three species.


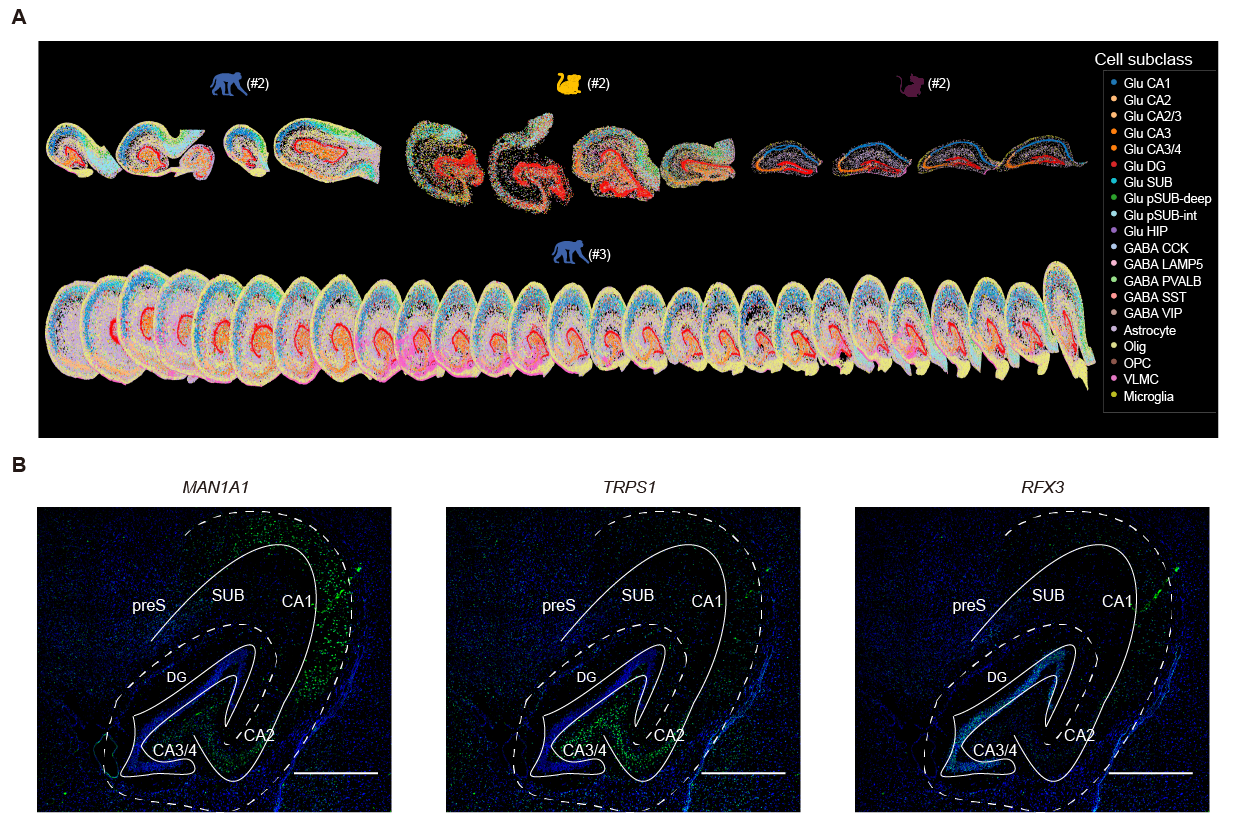


Figure S4. Transcriptomic similarity among glutamatergic cell types in CA2, CA3 and CA4

(**A**) Representative sections showing the spatial expression pattern of marker gene *RGS14* for CA2 glutamatergic cells in macaques and marker gene *COTL1* for CA2 glutamatergic cells in marmosets.

(**B**) Representative sections showing the spatial expression pattern of marker gene *EPHA6* for CA4 glutamatergic cells in macaques and marker gene *CARTPT* for CA4 glutamatergic cells in marmosets. The expression level was color-coded with scale bar shown at right.

(**C**) Dot plots showing the expression pattern of specific TFs for CA1, CA2, CA3, and CA4 glutamatergic cells in macaques, marmosets and mice. The size of the dot represents the proportion of the cells expressing indicated genes, and the color of the dot indicates the gene expression level.

(**D**) Dot plots showing the relative expression of genes involved in ion channel pathways across CA2, CA3, and CA4 glutamatergic cell types in macaques, marmosets, and mice. The size of the dot represents the proportion of the cells expressing indicated genes, and the color of the dot indicates the gene expression level. The dashed squares indicate the subunit genes that were different from those in CA3.

**(E)** FISH assay of *CARTPT* expression enriched in CA4 of a marmoset hippocampal replicate. Solid line, granule cell layer of DG; dashed line, pyramidal cell layer of CA3.

**(F)** Dot plots showing the expression pattern of gene module score of different iron channels for DG, CA1, CA2, CA3, and CA4 glutamatergic cells in macaques, marmosets and mice. The size of the dot represents the proportion of the cells expressing indicated genes, and the color of the dot indicates the gene expression level. Unpaired *t*-test for gene expression level, ***p<0.001.

**(G)** Summary of spike thresholds, peak amplitudes, and input resistance of CA3 vs. CA4 cells in mice. Spike thresholds (mV): -36.1±1.1 vs. -39.7±1.1, mouse. Peak amplitudes (mV): 42.8±1.7 vs. 36.7±2.0, mouse. Input resistance (MOhm): 124.0±32.14 vs. 92.8±14.8, mouse.

**(H)** Summary of spike thresholds, peak amplitudes, and input resistance of CA3 vs. CA4 cells in marmoset. Spike thresholds (mV): -42.4±1.3 vs. -39.8±1.2, marmoset. Peak amplitudes (mV): 42.6±1.9 vs. 40.9±1.9, marmoset. Input resistance (MOhm): 206.5±26.4 vs. 241.9±22.2, marmoset.

**(I)** Summary of spontaneous EPSC frequencies and amplitudes of CA3 vs. CA4 cells in mice. Frequency (Hz): 4.3±0.9 vs. 14.6±2.4, mouse; Amplitude (pA): 20.0±2.0 vs. 42.8±6.1, mouse. Unpaired *t*-test, *p<0.05, **p<0.01. Example recordings of spontaneous EPSC from pyramidal cells in CA3 and CA4 subregions of the mouse hippocampus.

**(J)** Summary of spontaneous EPSC frequencies and amplitudes of CA3 vs. CA4 cells in marmoset. Frequency (Hz): 4.6± 0.7 vs. 8.2±0.7, marmoset; Amplitude (pA): 33.4±3.5 vs. 60.2±7.6, marmoset. Unpaired *t*-test, *p<0.05, **p<0.01. Example recordings of spontaneous EPSC from pyramidal cells in CA3 and CA4 subregions of the marmoset hippocampus.


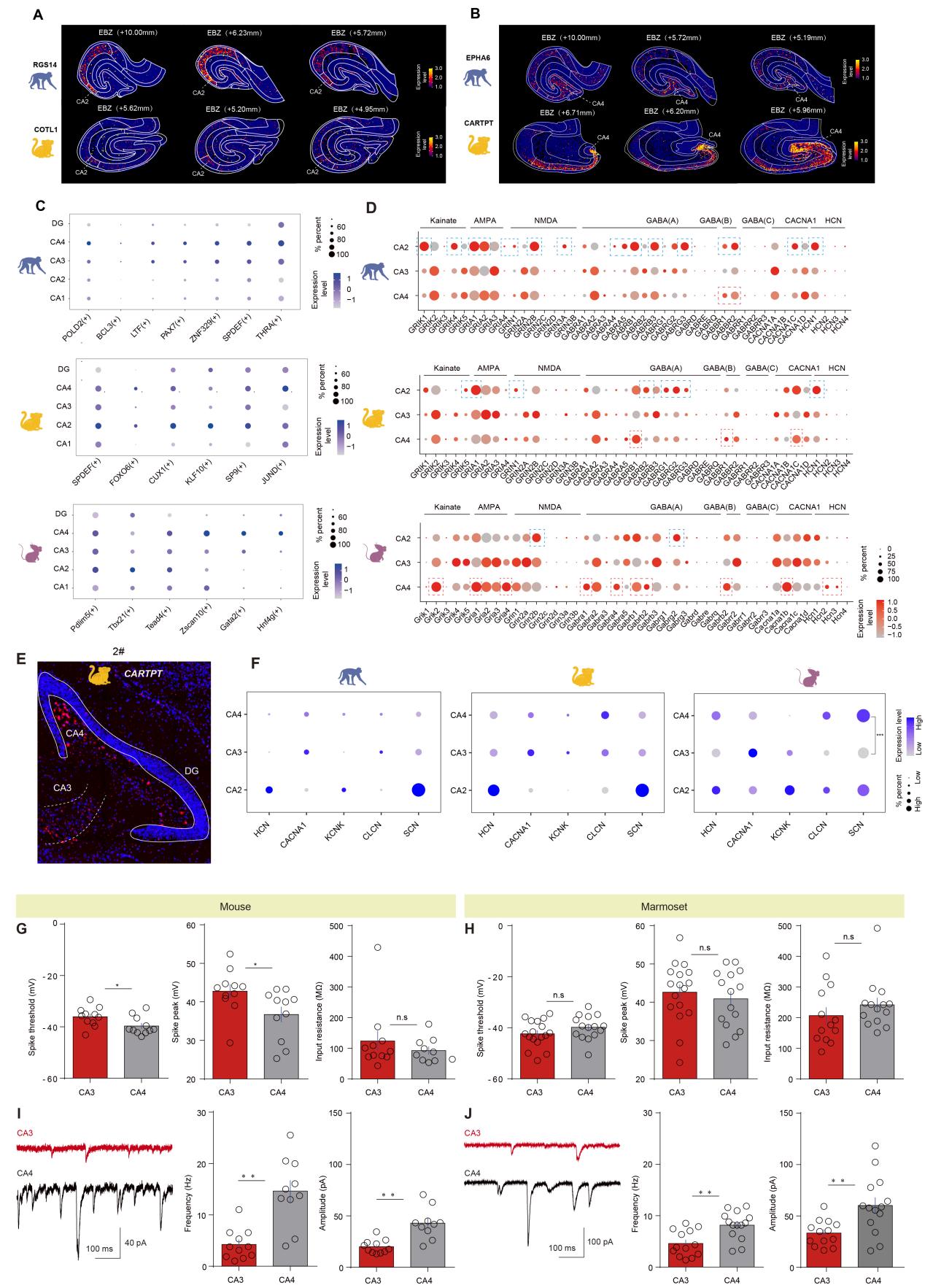


Figure S5. Molecular characterization of GABAergic cell types.

(**A**) The percentage of GABAergic cells among all neurons, and the percentage of five GABAergic subclasses (*VIP*, *SST*, *PAVLB*, *LAMP5*, and *CCK*) among all GABAergic cells in the hippocampus of six data datasets from four species. Published human datasets: human1^22^, human2 (GSE199243); human3 (GSE185553).

(**B**) UMAP embedding of GABAergic cells in the human (human1), macaque, marmoset and mouse hippocampus.

(**C**) The spatial distribution pattern of GABAergic cell types SST-1 and SST-2 in macaque sections at EBZ +12 mm, respectively.

(**D**) The gene expression pattern of *NPY* (a marker gene for cell type SST-1) was enriched in CA3/4 sections of macaques (upper) and marmosets (middle), but not mice (lower).

(**E**) The UMAP plot of integrated hippocampal GABAergic neurons in our macaque dataset and prefrontal cortical GABAergic neurons in published datasets^62^.

Scale bars, 1 mm.


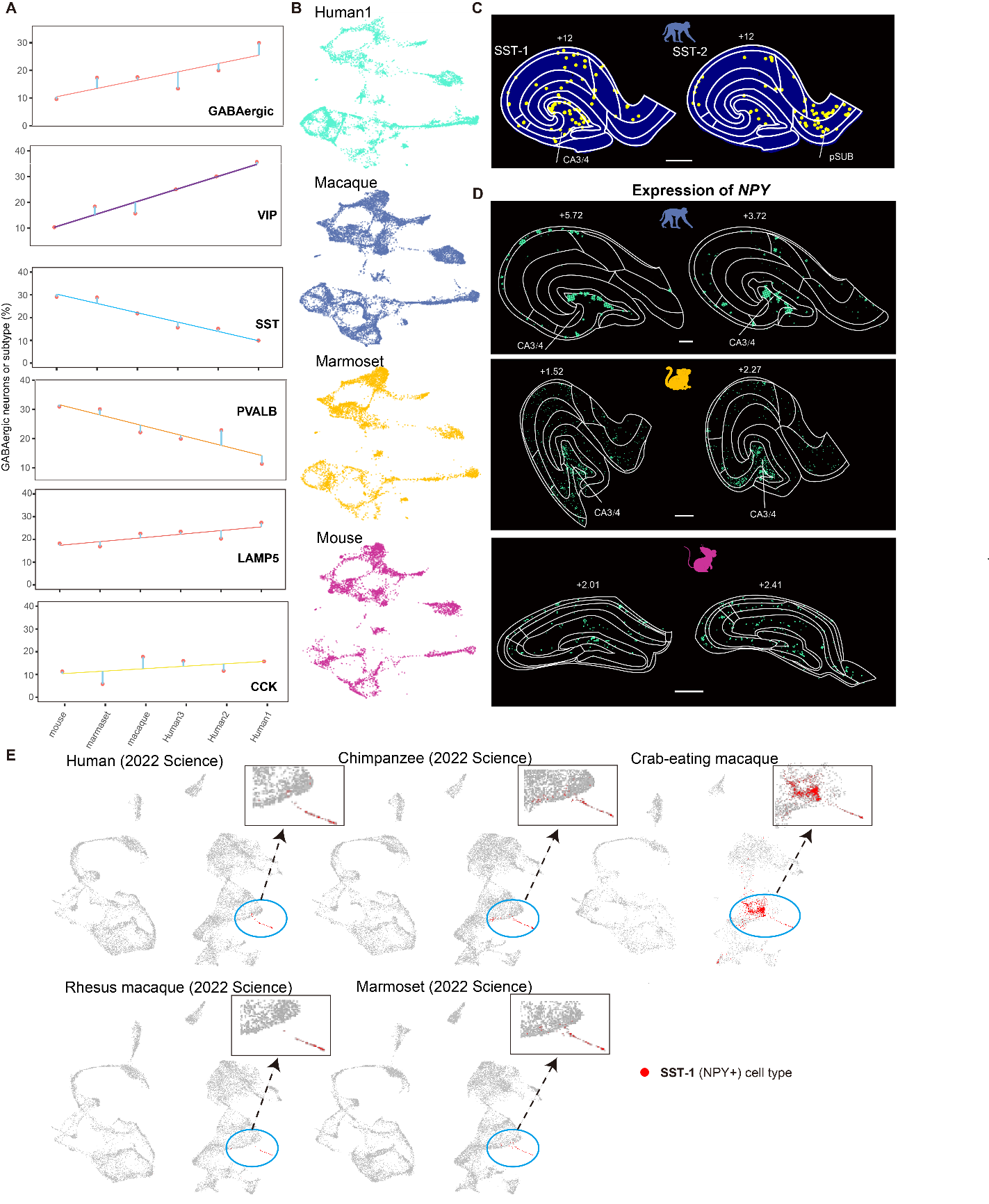


Figure S6. Molecular characterization of glial cell types.

(**A**) Unsupervised clustering analysis of four subclasses of glia cells.

(**B**) Stacked bar plots showing proportions of neurons (color-coded, legend shown at right) in spatial transcriptome-defined subregions for each glia cell type (at least 10 cells in each) in all three species. The subregion with high dominance (proportion>0.4) was marked with asterisk.

(**C**) Spatial distribution of four glia cell types in marmosets and mice, respectively. Scale bars, 1 mm.


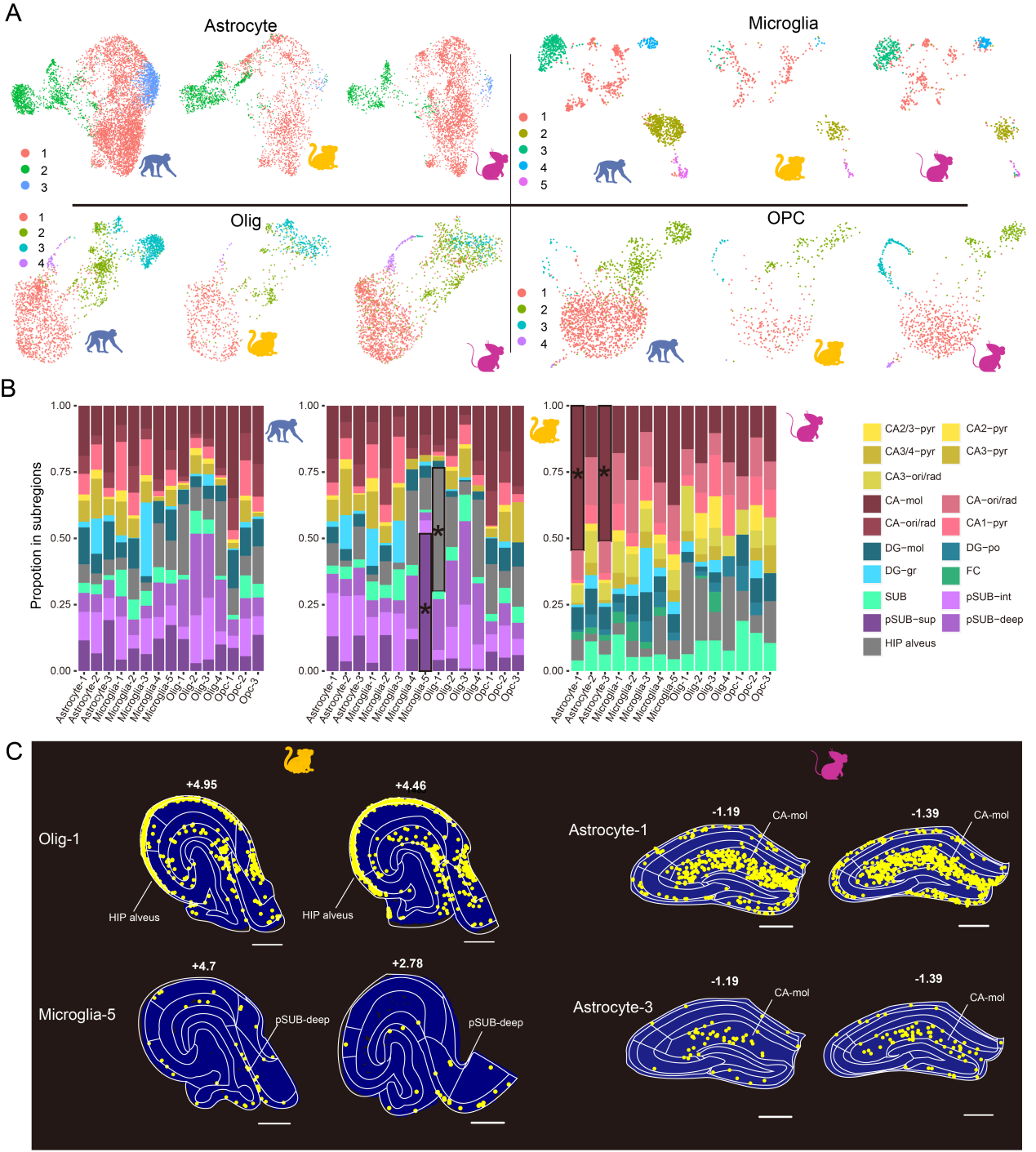


Figure S7. Molecular characterization of glutamatergic cell types

(**A**) UMAP visualization of pooled glutamatergic cell types in the macaque, marmoset and mouse hippocampus. Glutamatergic cells found only in macaques and marmosets were shown in red and classified as cell types such as “Glu pSUB-deep-1” (upper) and “Glu pSUB-int-1” (lower).

(**B**) Spatial distribution of primate-specific cell type Glu pSUB-int-2 in two representative hippocampal sections (EBZ coordinates shown above) from biological replicates of macaques. Red lines indicate the subregion pSUB-int.

(**C**) Dot plot showing the expression level of top 10 marker genes in Glu pSUB-deep-1 and the rest of glutamatergic cell types.

(**D**) Dot plot showing the expression level of top 10 marker genes in Glu pSUB-int-1 and the rest of glutamatergic cell types.

(**E**) Dot plot showing the expression score (calculated by the average expression of top 10 marker genes) of cell type Glu pSUB-int-2 in various hippocampal subregions of macaques, marmosets and mice.

(**F**) UMAP embeddings showing the integrative analysis of glutamatergic neurons from hippocampus and cortex of macaques.

(**G**) Heatmaps showing comparison of glutamatergic cell types between macaque hippocampus and cortex. The color intensity indicates the overlapping ratio between a hippocampal cell type and a cortical cell type in the integrated space.

(**H**) Top enriched pathways of highly expressed genes in primate-specific cell type Glu pSUB-int-2 in macaques and marmosets.

(**I**) Boxplot showing major depression disorder (MDD) scores between Glu pSUB-int-2 and other glutamatergic cell subtypes in macaques and marmosets. Wilcoxon rank sum test, *p < 0.05 for all.

(**J**) Heatmap showing the percentage of cells with various expression patterns of AMPA receptor subunit genes for each subicular glutamatergic cell type of macaques (left) and marmosets (right).


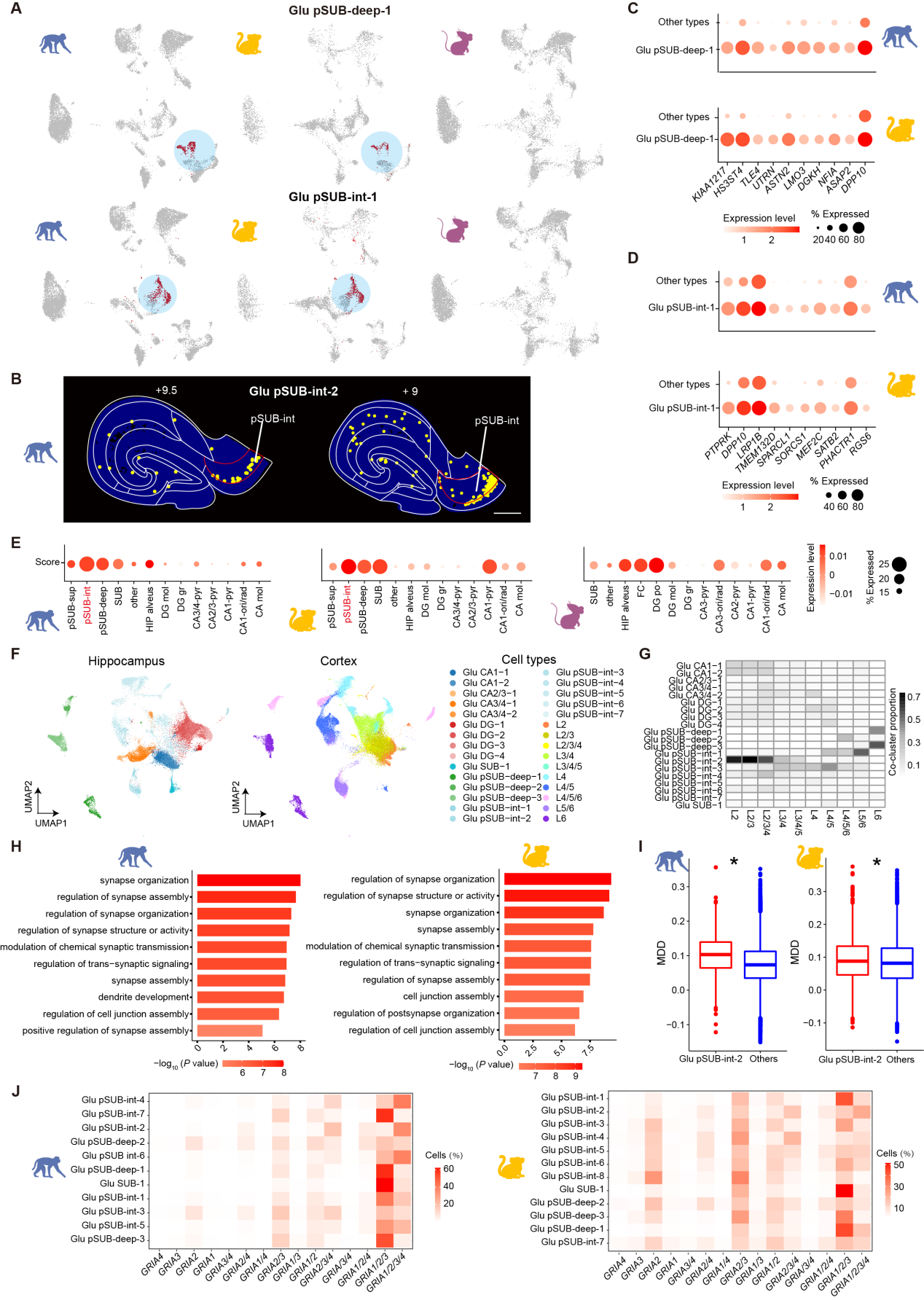


Figure S8. Comparative analysis of gene expression patterns of individual cell types

(**A**) Jaccard similarity of gene expression patterns in glutamatergic cell types of the hippocampus in macaques vs. marmosets (left) and macaques vs. mice (right). The grey level indicates Jaccard similarity with scale bar at bottom. Red squares, primate-specific cell types.

(**B**) Jaccard similarity of gene expression patterns in GABAergic cell types of the hippocampus in macaques vs. marmosets (left) and macaques vs. mice (right). The grey level indicates Jaccard similarity with scale bar at bottom.  Colored squares, GABAergic subclasses.


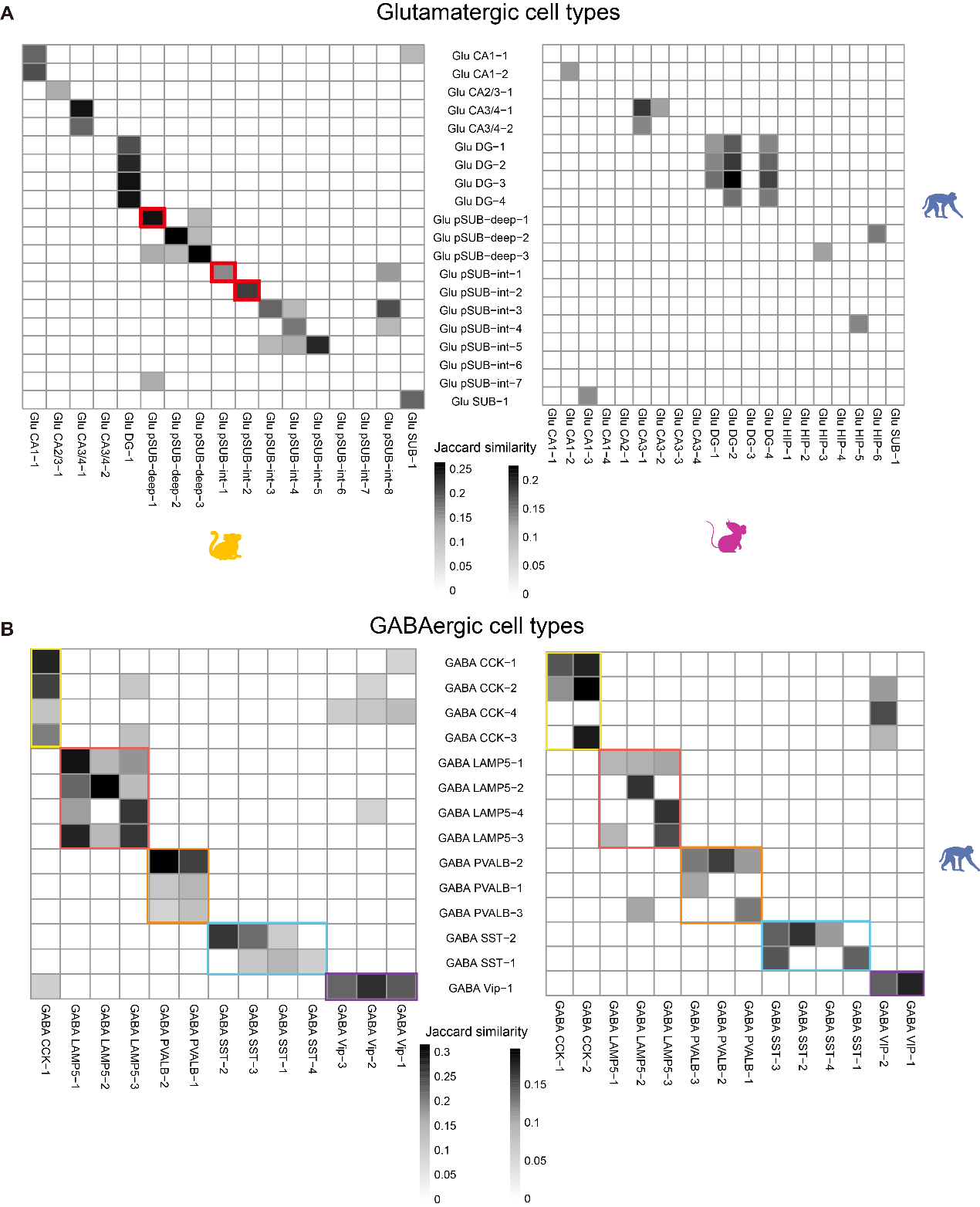


Figure S9. Integrative analysis of primate-enriched cell types with published mouse datasets

(**A**) The UMAP plot of integrated hippocampal glutamatergic neurons from our macaques and marmoset datasets and published mouse datasets, with subclasses annotated and color-coded.

(**B**-**D**) The same UMAP plot as in A. Glutamatergic cell types of “Glu pSUB-int-2” (B), “Glu pSUB-int-1” (C) and “Glu pSUB-deep-1” (D) were shown in red, which were only found in macaque and marmoset UMAP plots.

(**E**) The proportion of co-clustered cells that belong to the same cell type in macaque and mouse datasets was color-coded with scale bar shown on the right.


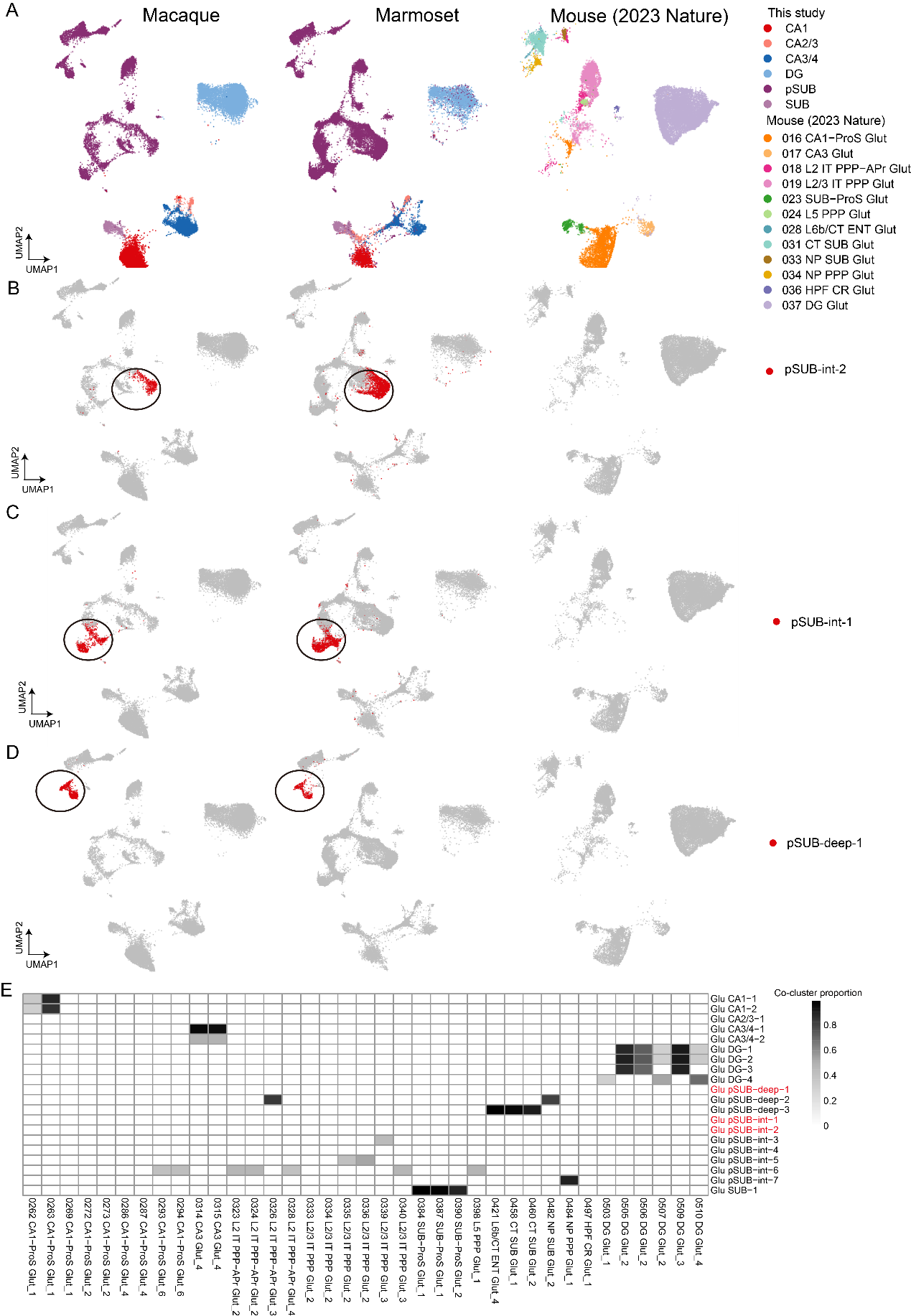


Figure S10. Integrative analysis of primate-enriched cell types with published human datasets

(**A**) Spatial distributions of glutamatergic, GABAergic and non-neuronal cell types along the longitudinal axis in macaques, marmosets and mice. The data of each species was shown with two columns: Left, the log of standard variation of the cell densities along the longitudinal axis for each cell subtype. The number of each cell type was color-coded with the scale bar shown above. Right, ridge plot showing the log of cell densities along the longitudinal axis. The anterior-preferring distribution was shown in red, whereas the posterior-preferring distribution was shown in blue.

(**B**) Spatial distributions and cell densities (color bar shown at right) of three primate-specific glutamatergic cell types in relatively anterior, intermediate, and posterior hippocampal sections from a macaque replicate (EBZ coordinates shown at the left side of sections). Subicular areas marked by red boxes were enlarged and shown at the right side of sections. Scale bars, 1 mm.

(**C**) Heatmaps illustrating the similarity of cell-type distribution along the longitudinal axis in macaques and marmosets.

(**D**) Bar plots showing the number of marker genes enriched in the synapse-related vs. unrelated functions for three glutamatergic cell types. The number of species-shared and species-specific marker genes was shown below for each cell type.

(**E**) Bar plot showing the enriched pathways of marker genes for Glu pSUB-int-4. Blue, red and green bars represent species-shared, macaque-specific and marmoset-specific pathways, respectively.

(**F**) Heatmaps displaying neighborhood enrichment scores of Glu pSUB-int-4 and Glu deep-1 with different cell subtypes along the anterior-posterior axis of the macaque and marmoset hippocampus. The neighborhood enrichment score was color-coded with scale bar shown at right. The grey color indicates that the score was not computable due to low cell number of paired cell types in that specific hippocampal section. Noted that the interaction between Glu pSUB-deep-1 and glia cells such as oligodendrocytes was strengthened from anterior to posterior sections in both macaques and marmosets.

(**G**) Dot plots showing the gradient expression of GABA receptors for Glu pSUB-int-2 cell type along the anterior-posterior axis of the macaque and marmoset hippocampus.


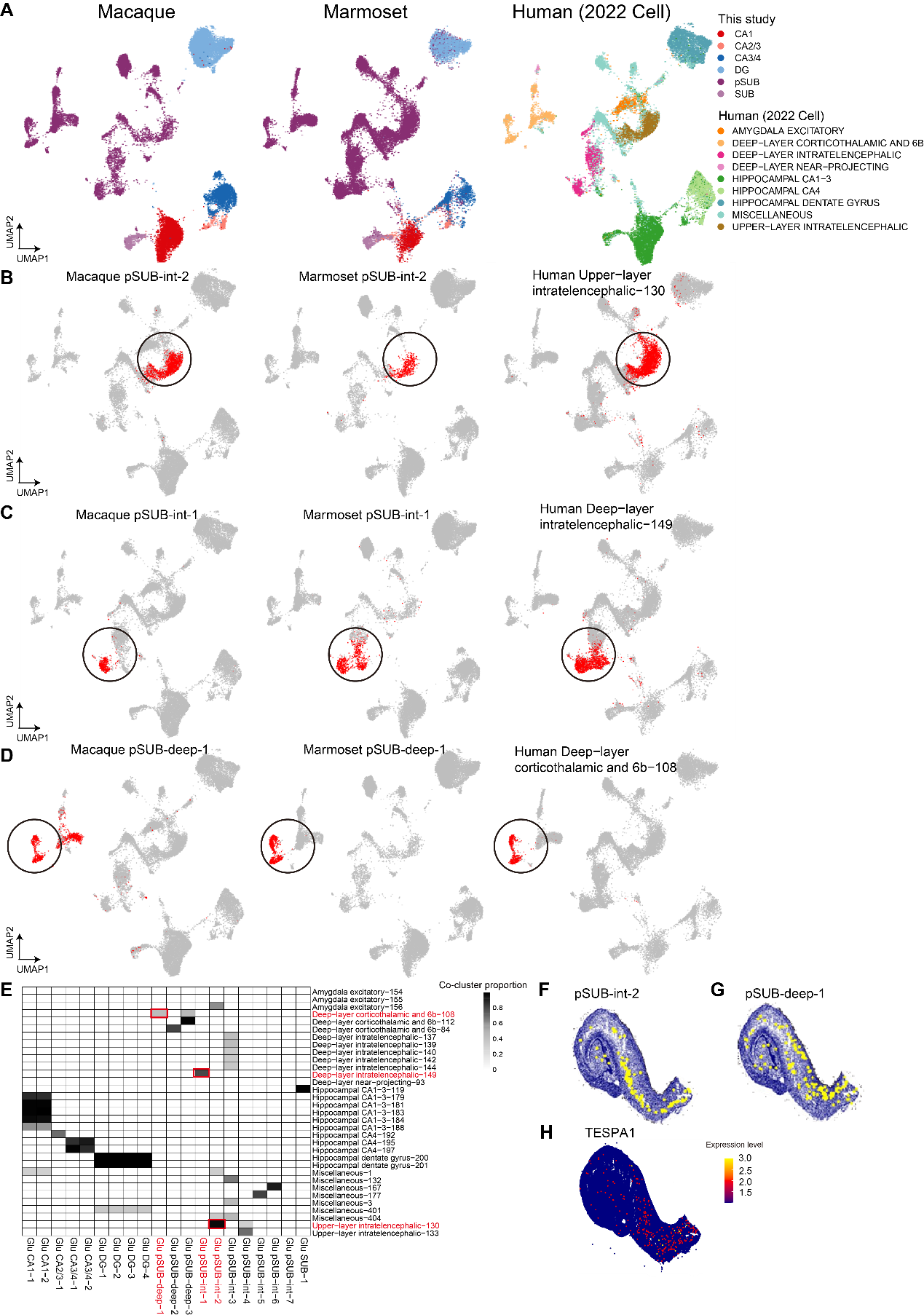


Figure S11. Longitudinal distribution of hippocampal cell types across species

(**A**) Spatial distributions of glutamatergic, GABAergic and non-neuronal cell types along the longitudinal axis in macaques, marmosets and mice. The data of each species was shown with two columns: Left, the log of standard variation of the cell densities along the longitudinal axis for each cell subtype. The number of each cell type was color-coded with the scale bar shown above. Right, ridge plot showing the log of cell densities along the longitudinal axis. The anterior-preferring distribution was shown in red, whereas the posterior-preferring distribution was shown in blue.

(**B**) Spatial distributions and cell densities (color bar shown at right) of three primate-specific glutamatergic cell types in relatively anterior, intermediate, and posterior hippocampal sections from a macaque replicate (EBZ coordinates shown at the left side of sections). Subicular areas marked by red boxes were enlarged and shown at the right side of sections. Scale bars, 1 mm.

(**C**) Heatmaps illustrating the similarity of cell-type distribution along the longitudinal axis in macaques and marmosets.

(**D**) Bar plots showing the number of marker genes enriched in the synapse-related vs. unrelated functions for three glutamatergic cell types. The number of species-shared and species-specific marker genes was shown below for each cell type.

(**E**) Bar plot showing the enriched pathways of marker genes for Glu pSUB-int-4. Blue, red and green bars represent species-shared, macaque-specific and marmoset-specific pathways, respectively.

(**F**) Heatmaps displaying neighborhood enrichment scores of Glu pSUB-int-4 and Glu deep-1 with different cell subtypes along the anterior-posterior axis of the macaque and marmoset hippocampus. The neighborhood enrichment score was color-coded with scale bar shown at right. The grey color indicates that the score was not computable due to low cell number of paired cell types in that specific hippocampal section. Noted that the interaction between Glu pSUB-deep-1 and glia cells such as oligodendrocytes was strengthened from anterior to posterior sections in both macaques and marmosets.

(**G**) Dot plots showing the gradient expression of GABA receptors for Glu pSUB-int-2 cell type along the anterior-posterior axis of the macaque and marmoset hippocampus.


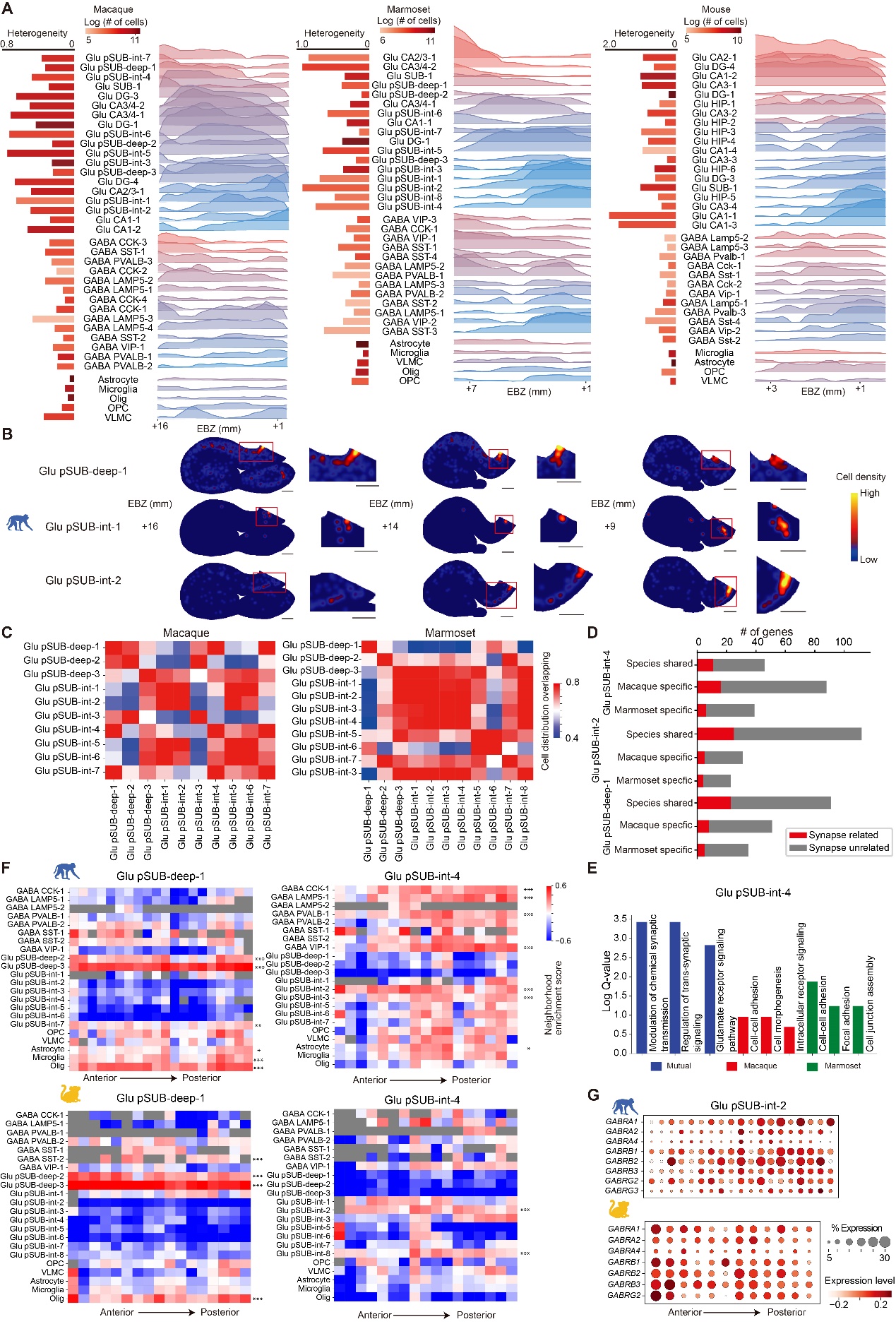


Figure S12. Cell-type and gene-expression distribution along the proximal-distal and superficial-deep axes

(**A**) Violin plots showing spatial distributions of glutamatergic cell types along the superficial-deep (left) and distal-proximal (right) axes in the macaque, marmoset and mouse hippocampus. Three dashed lines indicate the two ends and center of mass (CM) of *str. pyramidale*, respectively. *Distributions with offsets>0.5 (red text label in mice), **Distributions with offsets>0.75, ***Distributions with offsets>1.0 (red text label in primates).

(**B**) Heatmaps showing marker gene expression gradients along the superficial-deep (upper panels) and proximal-distal (lower panels) axes. In each panel, first and second 5 genes were marker genes with enriched expression in *str. pyramidale*. They exhibited opposite preferences (offsets>0.2) that were determined by spearman correlation tests. The normalized gene expression was color-coded with scale bar shown at right.

(**C**) Spatial expression patterns of marker genes enriched in superficial, deep, distal and proximal parts of the outlined subregion of “CA1-pyr”, respectively (from left to right in each row). Scale bars, 0.5 mm.


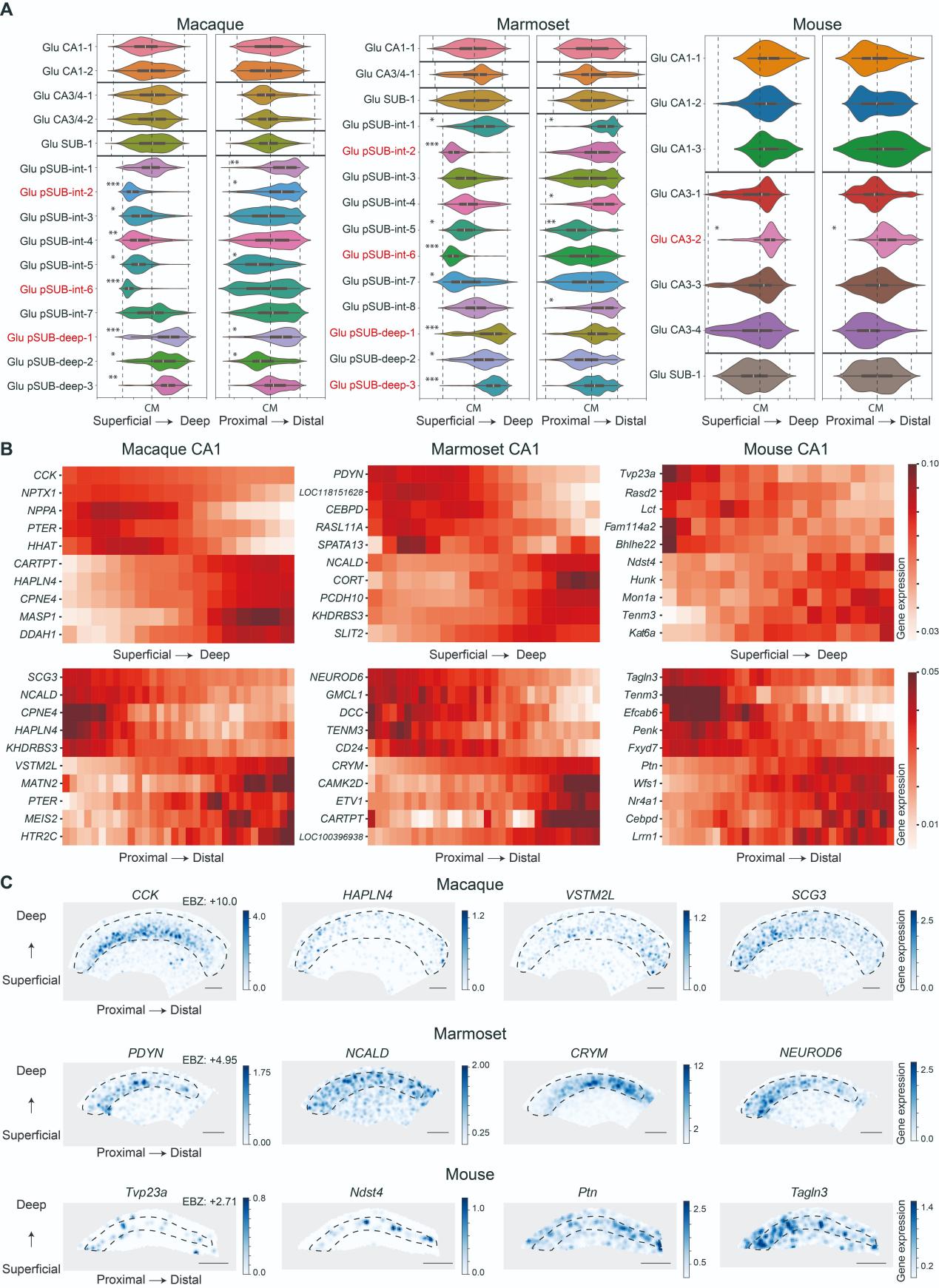

Supplement: nwaf595_Supplemental_Files [file nwaf595_supplemental_files.zip › Supplementary_Figures.docx]
